# Computer-aided diagnosis system for grading brain tumor using histopathology images based on color and texture features

**DOI:** 10.1186/s12880-024-01355-9

**Published:** 2024-07-19

**Authors:** Naira Elazab, Wael Gab Allah, Mohammed Elmogy

**Affiliations:** Information Technology Department, Faculty of Computers and Information, Mansoura University, 35516 Mansoura, Egypt

**Keywords:** Glioma computational pathology, Cancer grades, Color features, Texture features, Ensemble classifier

## Abstract

**Background:**

Cancer pathology shows disease development and associated molecular features. It provides extensive phenotypic information that is cancer-predictive and has potential implications for planning treatment. Based on the exceptional performance of computational approaches in the field of digital pathogenic, the use of rich phenotypic information in digital pathology images has enabled us to identify low-level gliomas (LGG) from high-grade gliomas (HGG). Because the differences between the textures are so slight, utilizing just one feature or a small number of features produces poor categorization results.

**Methods:**

In this work, multiple feature extraction methods that can extract distinct features from the texture of histopathology image data are used to compare the classification outcomes. The successful feature extraction algorithms GLCM, LBP, multi-LBGLCM, GLRLM, color moment features, and RSHD have been chosen in this paper. LBP and GLCM algorithms are combined to create LBGLCM. The LBGLCM feature extraction approach is extended in this study to multiple scales using an image pyramid, which is defined by sampling the image both in space and scale. The preprocessing stage is first used to enhance the contrast of the images and remove noise and illumination effects. The feature extraction stage is then carried out to extract several important features (texture and color) from histopathology images. Third, the feature fusion and reduction step is put into practice to decrease the number of features that are processed, reducing the computation time of the suggested system. The classification stage is created at the end to categorize various brain cancer grades. We performed our analysis on the 821 whole-slide pathology images from glioma patients in the Cancer Genome Atlas (TCGA) dataset. Two types of brain cancer are included in the dataset: GBM and LGG (grades II and III). 506 GBM images and 315 LGG images are included in our analysis, guaranteeing representation of various tumor grades and histopathological features.

**Results:**

The fusion of textural and color characteristics was validated in the glioma patients using the 10-fold cross-validation technique with an accuracy equals to 95.8%, sensitivity equals to 96.4%, DSC equals to 96.7%, and specificity equals to 97.1%. The combination of the color and texture characteristics produced significantly better accuracy, which supported their synergistic significance in the predictive model. The result indicates that the textural characteristics can be an objective, accurate, and comprehensive glioma prediction when paired with conventional imagery.

**Conclusion:**

The results outperform current approaches for identifying LGG from HGG and provide competitive performance in classifying four categories of glioma in the literature. The proposed model can help stratify patients in clinical studies, choose patients for targeted therapy, and customize specific treatment schedules.

## Introduction

Brain tumors are abnormal cell growths that occur within the brain. Brain tumors are classified into several categories based on their origin, rate of growth, and stage of progression [1, 2]. Tumors of the brain may be benign or malignant. Benign brain tumor cells seldom infect nearby healthy cells. They have defined borders and develop slowly (e.g., meningiomas and astrocytomas). Malignant brain tumor cells, such as oligodendrogliomas and astrocytomas of the higher grade, attack surrounding cells in the brain. They have fuzzy borders and spread rapidly. Primary brain tumors are classified as low-grade glioma (LGG) and high-grade glioma (HGG) [3].

Grades I and II are denoted as LGG, whereas grades III and IV are defined as HGG. Grades I and II are astrocytomas, grade III is an oligodendroglioma tumor, and grade IV is a glioblastoma multiforme (GBM). Astrocytoma and medulloblastoma are influenced in children. Oligodendroglioma, meningioma, and glioblastoma are affected in adults. GBM is an advanced stage of the brain tumor with few symptoms, which makes it difficult to classify. Early and accurate diagnosis of these malignancies improves patient survival [4].

Histopathology is the field of human tissue analysis for a specific disease. The procedure of examining human tissue in clinical practice is basically as follows. First, a biopsy is collected and sent to the pathology laboratory. The tissue is then stained on a glass slide in the lab. The aim of the stain is to highlight certain tissue features. For example, stained hematoxylin and eosin (H&E) tissue provides a dark purple color to the nuclei and rose for other structures. The pathologist can use a microscope to examine when the tissue is processed and stained [5]. Conventionally, pathologists analyze tissue sections under a microscope to diagnose or grade brain tumors. However, the manual diagnosis process is time-consuming and prone to human mistakes. Then, computer-aided brain tumor classification is widely needed [6].

Brain tumors must be detected accurately and early in order to be successfully treated. Brain cancer is one of the most lethal cancers for both men and women with low survival levels [7]. Early detection not only helps to develop better drugs but can also keep our lives in due time. Brain cancer is the leading cause of mortality in females aged 20 and under and males aged 40 and under [8]. According to studies, brain cancers are exceedingly heterogeneous, which is the fundamental challenge for brain tumor categorization and thus diagnosis [9].

Therefore, it is essential to diagnose tumors correctly because treatment depends largely on knowing the characteristics of the tumor and how it progresses. Tissue histopathology exposes the impact of cancer onset and progression at the sub-cellular level [10, 11].Histopathology images (HIs) are the principal information source for cancer diagnosis and prognosis. Digital microscope advancements have enabled the acquisition of high-resolution images of whole-slide tissue, increasing the use of virtual slides in histopathologic study [12].

**Fig. 1:**
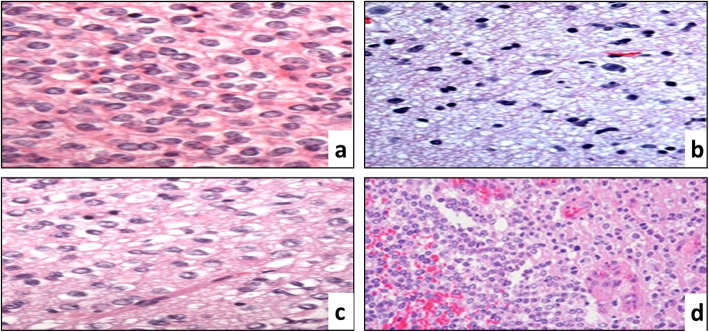
Glioma histopathological characteristics at 40x magnification: **a** Oligodendrogliomas are characterized by uniform appearing, **b** Astrocytoma consists of fibrillary neoplastic astrocytes, **c** Oligoastrocytoma contains a mixture of both tumor cell types, and **d** GBM contains astrocytic cells and new blood vessel growth

The pathological features of grades I-IV are illustrated in Fig. 1. All histopathological images in Fig. 1 are adapted from the cancer genome Atlas (TCGA) dataset [13]. Histopathology is the key approach to distinguishing GBM from LGG. In manual diagnosis, many HIs must be examined with various stains to diagnose a single instance, which is time-consuming [14]. Because of the increased complexity of HIs, accurate extraction of both visible and latent image features has become a more difficult challenge. Compared to traditional manual diagnosing techniques, computer-aided mechanisms produce better results [15].

It takes a lot of time and effort to manually diagnose and analyze brain histopathology images. Therefore, time and effort will be reduced by automated detection and diagnosis, assisting in the disease’s early detection. Diagnostic imaging’s popularity has resulted in a significant increase in pathologists’ workloads. The data explosion can be managed with Computer-aided diagnosis (CAD). With AI classifiers, the computational capacity of CAD systems can calculate various quantitative features to define tumor characteristics in real time and estimate tumor types and grades. Diagnostic processes can be sped up, and errors can be minimized with a quantitative approach.

This research aims to create computational ways to test the hypothesis that gliomas can be classified as LGGs or HGGs based on histopathology images. If the tumor grade is accurately diagnosed, this will aid in patient classification for therapeutic clinical trials. In this paper, quantitative feature extraction approaches and advanced machine learning methods were applied to analyze HIs. We assume that quantifying subtle histological characteristics extracted from HIS is instructive and leads to the stratification of LGG and HGGs with adequate sensitivity and specificity.

Statistical properties like entropy, contrast, correlation, homogeneity, and energy can be described using gray-level intensity features extracted from HIs. In medical image analysis, a statistical approach, specifically the gray-level co-occurrence matrix (GLCM) and gray-level run-length matrix (GLRLM) texture analysis method, are common. This paper applied a local binary pattern (LBP) for texture analyses to get useful information from the HIs. Extraction of features is particularly crucial when grading cancer with biopsy pictures. The brain cancer grading is often based on the morphologic, textural, nucleic, and color moment features.

GLCM has emerged as one of the most effective texture descriptors for use in image analysis among statistical methods. The standard GLCM algorithm, however, extracts features based on a pixel and its next neighboring pixel. Additional regional patterns on the image are irrelevant to it. Therefore, we apply a feature extraction method loacl binary GLCM (LBGLCM) that combines the LBP and GLCM algorithms that takes into account both the texture structure and the spatial information. The original GLCM’s limited capacity to capture texture data at various scales is another drawback. It is often assumed that texture information is fixed at a particular image resolution in descriptors. If differing scales are taken into account when extracting texture descriptors from the photos, the texture descriptors’ ability to discriminate between objects can be considerably increased. In order to generate a variety of picture representations of the original image, the pyramid decomposition method is used in this study to introduce a new approach for extending the LBGLCM to be more robust under scale variation.

This method concentrates on LBP,GLCM,LBGLCM, GLRLM,color histogram analyses and rotation and scale invariant hybrid image descriptor (RSHD) which based on the fusion of color and texture descriptor to process stained biopsies. Texture and color characteristics are particularly significant in examining the tissue image and give an image’s color intensity and distribution information. This study proposes a hybrid ensemble method for classification using a decision tree (DT), support vector machine (SVM) with the kernel of radial basis function (RBF), and the fast large margin based on the majority voting method.

The key contributions of this study can be summarized in the following points:

- Introducing enhanced classification method to classify brain tumors in more detail. It aims to classify tumors into more specific categories, such as astrocytoma, anaplastic astrocytoma, oligodendroglioma, and glioblastoma multiforme.
- The use of the rich phenotypes in histology images through multivariate pattern analysis approaches for glioma grade classification.
- Applying the most discriminative related feature extraction methods and combining these features in order to improve classification performance.
- The LBGLCM texture descriptor is extended to several scales based on a pyramid decomposition.
- The most distinguishing features are fed to a hybrid ensemble method for classification.
- To evaluate the proposed system, many performance measures are used. In addition, we validated our suggested system by comparing it to some existing systems.

The texture and color feature extraction techniques are used in a novel way in the suggested hybrid model, which has never been used to classify tumor grade from brain histopathology images before. This makes it distinctive from other hybrid models. To be more precise, we apply six different feature extraction methods-GLCM, LBP, multi-LBGLCM, GLRLM, color moment features, and RSHD-and combine the results to provide a more complete and accurate representation of the histopathological images.

The proposed method is predicated on the proposition that the combination of texture and color features can deliver complimentary information that can enhance the precision of tumor grade categorization. The intricate visual patterns that distinguish different tumor grades can be more effectively captured by combining these two kinds of characteristics. By sampling the image both in space and scale, we create an image pyramid that allows us to extend the LBGLCM feature extraction method to various scales. This method enables us to capture the multiscale properties of histopathology images, which can enhance the classification accuracy of brain cancer.

For the reader’s convenience, the used abbreviations in this paper are listed in Table 1. The rest of this paper is divided into four sections. Related work section reviews related work of the brain grade classification and the current weaknesses. The suggested framework is described in Materials, metrics, and software specification section. The proposed system section contains the proposed model’s experimental results, discussion, and comparisons. Experimental results section represents a conclusion and future work.

**Table 1:** The used abbreviations

HIs	Histopathology Images	MVP	Micro vascular of proliferation
LGG	Low grade glioma	PCA	Principal component analysis
HGG	High grade glioma	H&E	Hematoxylin and Eosin
GBM	Glioblastoma multiform	AUC	Area under the Curve
GLCM	Gray level co-occurrence matrix	ROI	Region of interest
GLRLM	Gray level run length matrix	CNN	Convolutional neural network
LBP	Local binary pattern	ACC	Accuracy
DT	Decision tree	WSI	Whole slide image
SVM	Support vector machine	PSD	Predictive sparse decomposition
RBF	Radial basis function	PCMD	Pixel-based color moment descriptor
ML	Machine learning	CLAHE	Contrast limited adaptive histogram equalization
DL	Deep learning	NMF	Non-negative matrix factorization
HOG	Histogram of oriented gradient	TPR	True positive rate
SFTA	Segments fractal texture analyses	PPV	Positive predictive value
RF	Random forest	SPC	Specificity
AANN	Adaptive artificial neural network	KNN	k-nearest neighbors
CAD	Computer-aided diagnosis	GLM	Generalized linear models
ROC	Receiver operating characteristic	DSC	Dice similarity coefficient

## Related work

Recently, various strategies for the automatic categorization of brain cancer have been proposed, which can be divided according to feature selection and learning mechanism into machine learning (ML) and deep learning (DL) technologies. In the field of medical imagery, ML algorithms played a crucial role. Since medical imaging techniques have become commonly employed in various medical applications, such as brain tumor detection and identification, researchers have concentrated on building automated systems for categorizing tumors with various medical imaging techniques.

The design of feature representation is an important direction for HIs. Manually produced features are very active research topic include fractal [16], morphometric features [17], textural characteristics [18], and object-like characteristics [19]. For example, Chang et al. [20] proposed morphometrically scarce tissue characteristics for the GBM [21] dataset and tumor, normal, and stromal for the KRIC [22] dataset at different places and levels to recognize the tumor, necrosis, and transition to necrosis. Because of the vast quantity of data, they additionally utilized multi-scale spatial pyramid matching. Kong et al. [23] classified glioblastoma tumors using DNA methylation-based techniques that used multi-modal medical images. Hand-crafted features, such as histograms of oriented gradient (HOG) and binary robust independent elementary features, were created for short local image descriptors where bag-of-patterns identified tumor regions. They also computed auto-encoder deep features for segmentation masks in tumor diagnosis.

The diverse and complicated forms, structures, volumes, and positions of brain tumor cells are considered some of the main problems for automatic tumor detection. A mix of the LBP, Gabor wavelet, and HOG patterns and segments-based fractal texture analyses (SFTA) is suggested by Amin et al. [24] to help prevent brain cancer. An unsupervised clustering technique that includes an integrated feature vector was the LBP mix of the cluster method. The random forest (RF) technique was utilized for 0.5 hold-out cross-validation to prevent over-alignment in complete, enhancing and non-enhancing regions with the projection and categorization of tumors. An alternative way to classify the brain tumor is developed using a modified level set method to segment the tumor area. The feature sets the invariant Gabor and time and the GLCM, which are extracted by employing a multilevel wavelet. The adaptive artificial neural network (AANN) is used to select brain tumor prediction functions after selecting the features. The whale optimization algorithm is used to improve the precision of the ANN for the layers of the network by Virupakshappa and Amarapur [25].

Barkerc et al. [26] examined the characteristics of the pathology images in coarse to fine ways. Spatial features, such as the shape of the tile region, color, and texture, are retrieved and classified into a clustering system. K-means are utilized to cluster the extracted features. The principal component analysis (PCA) is implemented to minimize the dimensionality and complexity of the data classification. Powell et al. [27] evaluated gliomas of low quality with a bag of words. The edge algorithm for detecting eosin stains and hematoxylin (H&E) is employed for nuclear segmentation. A global value is used to assign a threshold. The k-means technique is utilized for extraction features, and SVM is utilized to categorize total short-term and long-term patient survival rates. On the other hand,

Bhattacharjee et al. [28] gathered pathological imagery features in glioma patients and applied DL models. Different histological markers like pseudo-palisading necrosis, geographic necrosis, and inflammation, which led to an accuracy (ACC) of 90%, have been linked to overall glioma survival [29]. Histology traits enhanced glioma forecasting when paired with radiology characteristics [30]. Rathore et al. [31] intended to measure the predictive value of each feature type (imaging, clinical, and textural) and their combinations using a predictive classifier. Using the 10-fold cross-validation technique, the texture properties were correctly assessed with an average ACC of 75.12% and an area under the curve (AUC) of 65.2%. This study had various drawbacks. First, choosing the region of interest (ROI) area was semi-automatically delineated in digital pathology pictures, using a computer program to discover the ROIs and then an expert to check if every ROI was artifact-free. This method could be user-bias and time-consuming as well.

Hemanth et al. [32] suggested an approach that included a mean-field term within the usual objective function of the convolutional neural networks (CNN). The study proposed an automatic segmentation approach based on CNN, which determines small $3 \times 3$ kernels. They used the University of California, Irvine (UCI) dataset. Their method included the following levels: data collecting, preprocessing, average filtering, segmentation, feature extraction, and CNN via classification and identification.

The analysis pipelines of glioma cases were implemented in grades II, III, and IV by Wang et al. [33], employed H&E and ki-67 tissue-stained whole slide image (WSI). The pipeline comprises several processes: identification of the ROI, extraction of the picture feature, selection of features, automatic slide grading, and explanation of grading findings. Various image characteristics, such as nucleic forms and sizes and image density distribution, are calculated and sliced using the RF approach. The classifying process used an ML model for the best classification performance, automatically adjusting model parameters. The contribution of GLCM and GLRLM characteristics in the analysis and categorization of brain HIs was demonstrated by Durgamahanthi et al. [34]. The fundamental goal of this research was to examine the complex random field of cancer images using GLCM and GLRLM characteristics. These characteristics were utilized to distinguish between healthy and cancerous tissues. For classification, the SVM classifier with RBF kernel was applied.

Sikder et al. [35] proposed a model that uses supervised learning, CNNs, and morphological operations to segment, classify, and detect various cancer cell types from MRI and HIS. The cancer grades in this methodology are classified using CNN, and the cancer cells are divided using semantic segmentation. The system classified every image as malignant or not by using the pixel labels from the ground truth during training. Ahmad et al. [36] proposed classifying HIs for-breast cancer diagnosis based on transfer learning and DL. They used transfer learning to identify breast HIs on a minimal number of training photos without decreasing performance by applying the patch selection strategy. In order to extract features from the CNN, patches are initially retrieved from WSIs. The discriminative patches are chosen based on these features and then fed into an Efficient-Net model. An SVM classifier is also trained using features taken from the Efficient-Net architecture. Xiao et al. [37] proposed skeleton and lattice features, which are hand-crafted features. The vascular networks in the RCC histopathology images are accurately represented by these features in their geometric and topological characteristics. Then, using a variety of algorithms (both conventional and DL models), they created robust benchmark results on the VRCC200 dataset. On a second database VRCC60, which has 60 annotated images of the vasculature from 20 patients, we further demonstrate the benefit and robustness of the proposed characteristics.

Dasanayaka et al. [38] introduced a system that categorizes brain tumors into three groups, oligodendroglioma, glioblastoma, and astrocytomas. They applied a ResNet and DenseNet to classify WSI and a magnetic resonance imaging sequence. Prior to classifying the WSI, they used preprocessing approaches to obtain a low dimensional feature description for patched WSIs as suggested in [39]. Following preprocessing, patches with a size of 256 x256 pixels were used in the input WSI’s determined region of interest for the feature extraction step. With the help of a pre-trained ResNet-50 model, features are extracted. Each patch receives a feature vector from the ResNet model with dimensions of 1024X1, which is then sent to the classification stage, where a model with a series of densely linked layers is used. Attallah and Zaghlool [40] suggested an automated classification system to categorize the four subtypes of pediatric medulloblastoma brain tumors. They improved subtype identification using HIs from two hospitals by combining textural analysis with DL approaches. GLCM and GLRM were used to transform the original photos into textural images, which were then fed to three DL models (ResNet-101, Inception, and InceptionResNet). Additionally, they used the original photos to train these three DL models. From the models that were trained using both textural and original images, they were able to extract deep features.

Ker et al. [41] offered a Google Inception V3-based automated method for categorizing histological slides of breast and brain tissue. According to the study, brain histology samples can be classified as normal, LGG, or HGG. Additionally, the study unearths the benefits of transfer learning across various tissue types, which have not before been discussed. Rinesh et al. [42] used multiple techniques on hyperspectral images to analyze the localization of brain tumors. A mixture of k-based clustering techniques (k-nearest neighbor and k-means) is used to detect tumors, with the firefly algorithm being used to determine the ideal value of k. The requirement for manual calculation is decreased by this optimization method. A multilayer feedforward neural network is used for labelling after brain region segmentation.

For the processing of pathological imaging data, Zhou et al. [43] proposed the adaptive dual-branch network (ASI-DBNet). Local and global data are both captured by the ResNet-ViT parallel structure. ResNet and ViT branch communication is made easier by the adaptive sparse interaction block (ASIB). To eliminate extraneous data and improve the feature maps shared between the branches, ASIB has an attention mechanism built in. In addition to raising the caliber of the final feature maps, this improves the interaction’s effectiveness. Khan et al. [44] suggested an automated technique for classifying and detecting brain tumors that makes use of a saliency map and enhanced deep learning characteristics. The framework includes a saliency map-based tumor segmentation method, fusion-based contrast enhancement, feature extraction from the average pooling layer, entropy serial fusion, feature selection using a dragonfly optimization algorithm, and classification using an extreme learning machine.

Syedsafi et al. [45] suggested a two-step approach for analyzing brain tumors. T2-weighted MRI images are first divided into tumor and normal tissue categories. An SVM classifier trained on 8x8 image block features extracted with the GLCM is used in this classification. The process then uses a color-based segmentation technique to separate FLAIR and T1-weighted Contrast-enhanced MRI images. Multi-scale morphological texture features and least squares SVM are powerful tools used by Khan et al. [46] .

Gül and Kaya [47] examined a dataset of brain images that included scans from both tumor and healthy patients. They created a two-stage method after being motivated by the effectiveness of hybrid models in medical image analysis. Image enhancement was the main focus of the first phase. They made use of three different LBP variations: step-LBP, angle-LBP, and the conventional LBP. These algorithms successfully encode the images’ local textural information. Using a battery of eleven classification algorithms, they assessed the improved image features that the LBP variants had extracted in the second stage. They discovered four particularly successful classification algorithms: random forest (RF), optimized forest, rotation forest, and instance-based learner, through a rigorous selection process based on experimental performance.

Nanda et al. [48] presented the Saliency-K-mean-SSO-RBNN classification model, which combines the salience-K-mean segmentation technique with the social spider optimization algorithm in the Radial Basis Neural Network. For segmenting the tumor region, a hybrid saliency map with K-means clustering is used. Next, features are extracted from the segmented image using the multiresolution wavelet transform, Kurtosis, Skewness, inverse difference moment, and Cosine transform. Table 2 provides an overview of some recent related work.

**Table 2:** A summary of some current related work

Study	Analysis Type	Methodology	Dataset	Performance
Saba et al. [49]	Brain tumor detection	Utilizing integration between handcrafted and DL features	MICCAI challenge databases	ACC = 91.7%
Rathore et al. [31]	LGG classification	Training of SVM models with linear configuration was made with the texturing features.	TCGA	ACC= 90.5%
Mousavi et al. [50]	Discrimination of LGG and HGG	The cell segmentation and the generation of the cell count profiles for identification of the pseudo palisading necrosis were developed. A hierarchical decision is made via a DT mechanism.	TCGA	ACC= 89.7%
Wang et al. [33]	Automated glioma grading	Visual factors with first and second-order features were retrieved, including morphological and sub-visual factors.	Shandong Provincial Hospital affiliated to Shandong University	ACC= 90%
Khan et al. [44]	brain tumor detection and classification	Using a saliency map and deep learning feature optimization	BraTs2018, 2019, 2020	Acc= 95}
Zhou et al. [43]	Grading of Brain Cancer	The ASI-DBNet which is a ResNet-ViT dual-branch network with adaptive sparse interactions	N/A	Acc= 95.2%
Ker et al. [41]	brain histology classification	using the Google Inception V3	Private dataset from the Department of Pathology at Tan Tock Seng Hospital, Singapore	prescion= 98%

According to the prior review of the present literature on the ML methods, the most significant restrictions in the diagnosis of brain grades from histology images can be summarized in the following points:

- Very small number of the previous studies implemented multi-classification for the brain tumors from HIs.
- The texture images include useful diagnostic information, essential for processing medical images and discriminating between benign and malignant cancers. However, these traits are insufficient to identify and distinguish between all grades. Other reliable features must be identified and discriminated.
- Some researchers have suggested a diagnosis of brain tumor grades. These models were conservative, inefficient, and imbalanced in the real world.
- Some studies rely on user bias, and it is time-intensive.
- Another major constraint of certain studies is the use of retrospective data.

In order to overcome these restrictions, we performed all the necessary phases to analyze the biopsy, extract characteristics, and classify the images of brain tissue that worked well with promising results. Automated computerized cancer classification systems will assist pathologists in diagnosing and categorizing cancer. We were able to collect helpful information from HI using ,GLCM,GLRLM, LBP, and multi-LBGLCM to recognize cancer grades in biopsy images. The color moment method can also be utilized to extract the various color information types found in histology biopsy images. A color histogram has been implemented in the current work to visualize tissue image color variation. Our work reveals that texture descriptor at multiple scale , pixel-based color moment descriptor (PCMD), and RSHD which based on fusion between color and texture descriptor can be implemented to extract significant characteristics in histological brain cancer images. In addition, none of the above approaches worked on hybrid ensemble classifying systems, which enabled to produce good evaluation by utilizing traditional classifiers since the best properties of each classifier combined to provide good results.

## Materials, metrics, and software specification

This section includes a description of the dataset, hardware and software specifications, and evaluation measures. This section contains comprehensive details about the dataset that used in the study. This dedicated section provides a detailed explanation and discussion of the performance assessment metrics used to evaluate our methodology.

### Datasets description

The TCGA [13] provided a set of 821 whole-slide pathology images of individuals with glioma (median age: 49.65 years, male: 427, female: 308, median survival: 761.26 days). TCGA is comprised of two brain cancer types: LGG and GBM. The data collection for LGG covers tumors of grade II and grade III. All 821 (506 GBM, 315 LGG) pictures of TCGA, with a complete pathologic and molecular data complement, have been selected to test the performance of the proposed system. The dataset is described in Table 3.

**Table 3:** The characteristics of TCGA dataset

Characteristics		Complete Dataset	Grade-II	Grade-III	Grade-IV
No. of patients		821	108	207	506
Median overall survival		761.26	1128	842.23	532.45
No. of deaths		516	25	89	390
Age		49.65	40.37	45.68	56.79
Gender	Male	513	64	138	311
	Female	308	44	69	195

### Hardware and software specifications

The proposed system is implemented using pycharm 2021.1.1 with Keras, Pandas, Itertools, Numpy, Sklearn, and Matplotlib libraries. The system is run on a machine with 16 GB RAM and Intel(R) Core (TM) i7/4.5 and an NVIDIA GeForce GTX with 4 GB VRAM.

### Evaluation metrics

We used five measurements to evaluate the proposed framework’s performance. These measurements include ACC, true positive rate (TPR)/sensitivity, positive predictive value (PPV)/precision, and specificity (SPC). The grading phase is assessed using the Dice similarity coefficient (DSC). The DSC calculates the relationship between two fields in terms of true/false positive and negative values. The greater the DSC, the more precise the stage of tissue classification. DSC provides a unified understanding of the Precision and Recall measurements. It represents the precision and recall harmonic mean. This basically suggests that a high DSC cannot exist without high precision and recall. A model is doing well all around when its DSC is high. DSC should be included with Sensitivity and Specificity for technique comparability.

1 $$\begin{aligned} ACC = \frac{TP + TN}{TP + TN + FP + FN} \end{aligned}$$

2 $$\begin{aligned} TRP = \frac{TP}{TP + FN} \end{aligned}$$

3 $$\begin{aligned} PPV = \frac{TP}{TP + FP } \end{aligned}$$

4 $$\begin{aligned} SPC = \frac{TN}{TN + FP } \end{aligned}$$

5 $$\begin{aligned} DSC = \frac{2 TP}{2 TP + FP + FN} \end{aligned}$$

## The proposed system

**Fig. 2:**
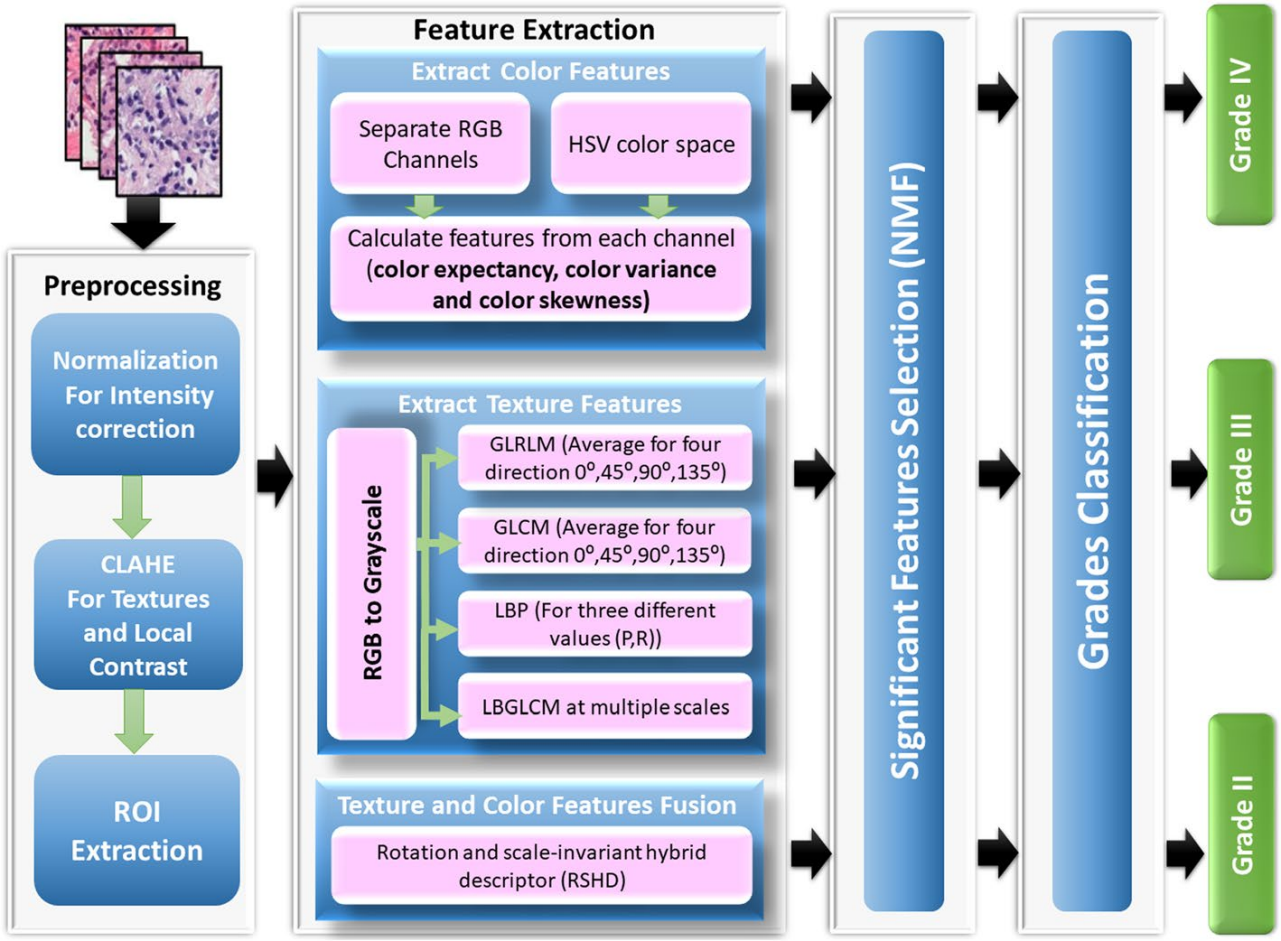
The proposed system for classifying brain grades from digital pathology images

A combination of features collected from images of tissue samples and microscopic biopsies was used for a hybrid ensemble classification using RBF-SVM, DT, and a fast large-margin classifier. In order to extract texture, we employed the LBP feature vector and computed GLCM and GLRLM. For brain cancer grading, color-feature extraction has also been done. These characteristics are appropriate for the various types of pathology for the image analysis selected for the research. The hybrid ensemble classification has been utilized to classify different brain cancer grades. The methodology provided in Fig. 2 illustrates how color and texture features are extracted from an image and how the hybrid ensemble classification is performed utilizing the relevant characteristics. It displays the proposed system architecture, consisting of four processing steps. First, the preprocessing phase is applied to minimize noise, remove light effectiveness and enhance the HI contrast. Second, the feature extraction phase is performed to obtain several important features from HI. Third, the reduced feature phase is used to lower the number of features analyzed, decreasing the time the system is calculated. The grading stage has finally been developed to determine different brain cancer grades. This strategy is predicted to enhance the ACC rate of classification compared to the method presented in the previously published research. The phases of the proposed system are described in detail in the following subsections.

### Data preprocessing

Due to variations in stain production processes, staining procedures used by different labs, and color responses produced by various digital scanners, images of tissues stained with the same stain exhibit undesirable color fluctuation. The H &E stain, which surgical pathologists utilize to expose histological detail, is a good example. The natural dye hematoxylin, produced from logwood trees, is difficult to standardize across batches since it is prone to precipitation while in storage, which can lead to daily variations even within a single lab. Stain normalization is an important step since the diversity in scan and handling circumstances. This technique reduces tissue sample variation. The uniformity of stain intensity has been proven to be essential in creating methods for the quantitative analysis of HIs in earlier investigations [51]. This paper presents the improved image enhancement method based on global and local enhancement. Two key steps comprise the suggested technique. First, the log-normalization function, which dynamically changes the image’s intensity contrast, encounters the raw image intensity adjustment. The second technique, called contrast limited adaptive histogram equalization (CLAHE), is used to improve the photos’ local contrast, textures, and microscopic features. After the two steps of normalization have applied, the ROI was extracted.

In addition to reducing the light reflection impact, the preprocessing phase is implemented to enhance the contrast of the RGB images ($g_{n}$). The CLAHE approach with $8 \times 8$ tiles was applied. CLAHE divides the image into predefined-size tiles. The contrast transform function is then computed separately for each tile. Finally, it merges adjacent tiles with bilinear interpolation to prevent artifacts from occurring in borders. The contrast-enhanced image ($g_{n}$) is fed into the next stage, which extracts features from the HIs of the brain. After two normalization steps, the approach automatically detects the ROIs based on the amount of nucleus in regions to reflect cell proliferation. The HIs were initially split into tiles in order to permit the process of high-resolution imaging with a resolution of $512 \times 512$ pixels [52]. Then, we found the five nuclear tiles of the highest density were classified as ROIs, using the watershed nuclei identification algorithm for each tile [53].

### Feature extraction

In order to extract texture and pixel-based color moment features, the two-dimensional (2D) GLCM and color-moment approaches were applied. Texture characteristics were extracted from LBP and GLCM, and colors from grayscale images were recovered from the original color image on three channels.

#### Texture features

**Statistical Method:** GLCM is a statistical approach that evaluates texture based on pixel space. To assess the texture of the image, we generated a GLCM. We then extracted statistical measurements from the matrix to determine how many pairs of pixels with certain values were found in a defined spatial relation between them. For feature extraction, the following directions were provided for the co-occurrence matrix: $0^{o}$ [0, 1], $45^{o}$ [-1, 1], $90^{o}$ [-1, 0], and $135^{o}$ [-1, -1].

Always within range of [0, 1] are the GLCM matrix. For texture analysis, the size of the GLCM matrix for sub-images at the first and second levels were $128 \times 128$ and $64 \times 64$. The GLCM calculates six different texture forms: contrast, homogeneity, correlation, energy, entropy, and dissimilarity. The following formulae were used to calculate these characteristics:

6 $$\begin{aligned} Contrast = \sum \limits _{i=0}^{N-1} \sum \limits _{j=0}^{N-1} |i- j|^{2} \rho (i-j) \end{aligned}$$

7 $$\begin{aligned} Homogeneity = \sum \limits _{i=0}^{N-1} \sum \limits _{j=0}^{N-1} \frac{\rho (i-j)}{1+ (i+J)^{2}} \end{aligned}$$

8 $$\begin{aligned} Correlation = \sum \limits _{i=0}^{N-1} \sum \limits _{j=0}^{N-1} \rho (i-j) \frac{(i-\mu _{x})(j-\mu _{y})}{\sigma _{x} \sigma _{y}} \end{aligned}$$

9 $$\begin{aligned} Energy = \sum \limits _{i=0}^{N-1} \sum \limits _{j=0}^{N-1} \rho (i-j)^{2} \end{aligned}$$

10 $$\begin{aligned} Dissimilarity = \sum \limits _{i=0}^{N-1} \sum \limits _{j=0}^{N-1} |i-j| \rho (i-j) \end{aligned}$$

where $p(i - j)$ is the probability matrix co-occurrence, separated by a specified distance, to combine two pixels with intensity (*i*, *j*). *N* denotes the grey level of quantization, and the means for row *i* and column *j*, respectively, are $\mu _{x}$ and $\mu _{y}$, and $\sigma _{x}$ and $\sigma _{y}$ are the standard deviations, within the GLCM.

GLRLM is a texture analysis method that works especially well with grayscale images. The patterns created by pixels with the same intensity value (gray level) are statistically quantified. GLRLM has been widely used in a variety of medical imaging modalities, including histopathological images, to analyze tissue microarchitecture and detect subtle abnormalities that are not visible to the naked eye. GLRLM does more than just analyze pixel intensities; it meticulously tracks how many pixels of the same intensity line up in a row, generating a map of these "runs" and their lengths. This is an overview of its features:

- **Gray Level:** A grayscale image’s pixel intensity, which ranges from 0 (black) to 255 (white), with intermediate values denoting various shades of gray.
- **Run Length:** The image is scanned in multiple directions ( $0^{o}$, $45^{o}$, $90^{o}$, and $135^{o}$ ). A run is a collection of neighboring pixels that have the same grayscale. The number of consecutive pixels with that specific intensity is referred to as the run’s length.

By analyzing features extracted from this map, GLRLM provides a quantitative description of the image’s texture, which is useful for a variety of applications, particularly in medical imaging, where distinguishing subtle textural variations is critical for accurate diagnosis. The capacity of GLRLM to extract texture features associated with the linear or elongated structures seen in images-structures that might be suggestive of specific pathological traits-is one of its primary advantages.

A wide range of features that perfectly capture the texture of the image are extracted by GLRLM. These characteristics, such as Long Run Emphasis (LRE) and Short Run Emphasis (SRE), indicate the frequency of densely populated or large areas with comparable intensities. Furthermore, Run Length Non-uniformity (RLN) investigates the variation in run lengths itself, while Gray Level Non-uniformity (GLN) explores the variety of shades within runs. Together with other features, these help to provide a comprehensive picture of the texture of the image, which is important for brain tumor classification, where minute differences in texture can make a diagnosis more accurate. The following equations were used to calculate this features:

11 $$\begin{aligned} SRE = \frac{ \sum \nolimits _{i=1}^{Ng} \sum \nolimits _{j=1}^{Ng} p(i,j) }{ 1 + (i+j) } \end{aligned}$$

where Ng: The number of gray levels in the picture; p(i,j): The normalized value at the GLRLM matrix’s position (i,j).

12 $$\begin{aligned} LRE = \frac{ \sum \nolimits _{i=1}^{Ng} \sum \nolimits _{j=1}^{Ng} p(i,j) }{ 1 + |i-j| } \end{aligned}$$

13 $$\begin{aligned} GLN = \sum \limits _{i=1}^{Ng} \sum \limits _{j=1}^{Ng} p(i,j) \cdot (i - \mu _g)^2 \end{aligned}$$

where $\mu _g$ represents the image’s mean gray level.

14 $$\begin{aligned} RLN = \sum \limits _{i=1}^{Ng} \sum \limits _{j=1}^{Ng} p(i,j) \cdot (j - \mu _r)^2 \end{aligned}$$

where $\mu _r$ represents the average run length computed in all directions.

**Figure:**
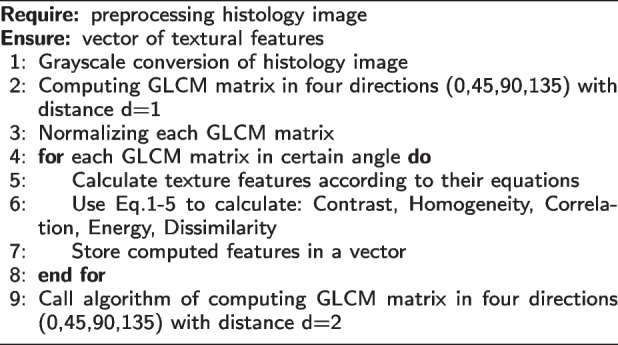
**Algorithm 1** Calculate GLCM texture feature

**Figure:**
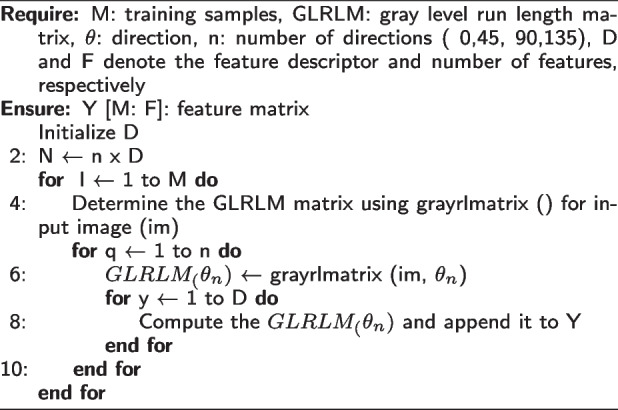
**Algorithm 2** Calculate GLRLM texture feature

**Local Binary Pattern:** LBP is a basic but strong local texture descriptor that considers each pixel’s center-value neighborhood, and the result is represented as a binary code. Suppose having a grayscale image consisting of a 3x3 pixel block, with X representing the central pixel and its eight neighbors:

$$\begin{aligned} \qquad \qquad \qquad \qquad \quad \begin{array}{ccccc} * & * & * & * & * \\ * & 120 & 130 & 140 & * \\ * & 110 & \text {X} & 130 & * \\ * & 60 & 70 & 80 & * \\ * & * & * & * & * \\ \end{array} \end{aligned}$$

Assume that the central pixel (X) has a gray level of 100. We’ll take a clockwise comparison with each of its neighbors. A neighbor’s binary code position is assigned a "1" if its intensity is greater than or equal to the central pixel. If not, a "0" is put in place. The binary string in this case, according to clockwise order, is 11001001.

Finally, the LBP feature value is obtained by converting this binary string to decimal. The decimal value in this instance is 201.To compute LBP features, this process is repeated for every pixel in the image patch. Following that, the LBP feature values can be utilized for additional analysis, like segmentation [54] or texture classification [55].

In a neighborhood (*P*, *R*), the fundamental LBP values are calculated by Eq. 15.

15 $$\begin{aligned} LBP_{P,R}= \sum \limits _{P=0}^{P-1} S(g_p - g_c)^{2^P} \end{aligned}$$

The intensity value ($$g_{c}$$) is identical to the density of the pixel in the center of the local district, whereas ($$g_{p}) =(0,1, ..., P-1)$$ is the grey value of *P* pixels with a R $$(R > 0)$$ creating a set of neighbors with a circular symmetry. In this study, another LBP extension with a reduced feature vector for the rotation of invariant regular modes is used [56]. This paper uses the LBP extension, which is explicitly familiar with heterogeneity by weighting local texture patterns. The second moment (variance) of local districts is used to expand LBP histograms with information on heterogeneity to better hold the polymorphism in histopathological pictures. The texture characteristics are extracted from an area of circular pixels with p-members and radius *R*, which were referred to as (*P*, *R*).

The LBP characteristics and heterogeneity measures have been retrieved for three different values (*P*, *R*) to take advantage of multi-resolution analysis. In addition, we studied two different, albeit related, techniques to detect heterogeneity:

1. Second moment (variance) of the average of the neighborhood, as presented by Eq. 16.
  16 $$\begin{aligned} V = \frac{1}{p} \sum \limits _{i=1}^{p} (g_{i} - \mu )^{2} \end{aligned}$$
2. Local dissimilarity computed based on any concept of homogeneity capturing a set of elements uniformity. When every element has the same value, the homogeneity of this set is the same. Local homogeneity *H* is calculated as by Eq. 17 [57].
  17 $$\begin{aligned} H = 1 - \frac{1}{L} \sqrt{\sum \limits _{i}\sum \limits _{j} (w_{ij} - m)^{2}} \end{aligned}$$
  where $w_{ij}$ are regional pixels, *m* is the mid or middle value in the region of the pixels, and *L* is the area size.

**Fig. 3:**
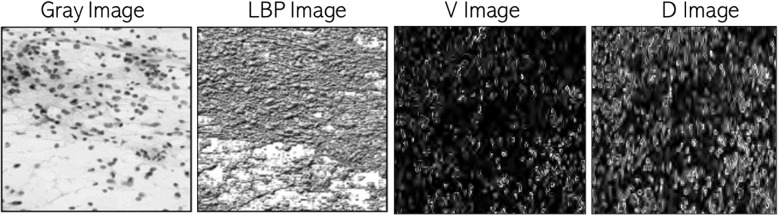
Image examples of LBP and heterogeneity based on principles of variance and dissimilarity

Figure 3 shows heterogeneity images in the first row based on the notions of variance and differences for an intuitive comprehension of the suggested method.

**Figure:**
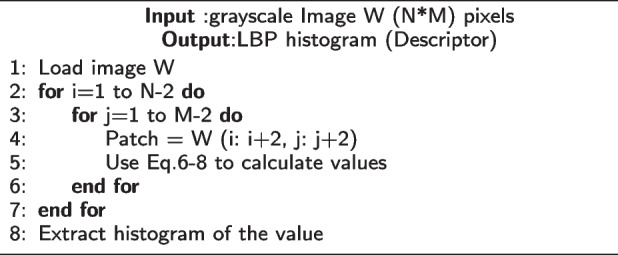
**Algorithm 3** Calculate LBP texture feature

**Local Binary Gray Level Co-occurrence Matrix (LBGLCM):** LBP and GLCM are combined to create the LBGLCM method. The LBP operator is initially applied to the raw image for this process. The LBP operator is used to analyze the image and produce a texture image. Finally, the resultant LBP image’s GLCM characteristics are retrieved. When extracting the features, the Traditional GLCM algorithm bases its operation on a pixel and its subsequent neighbor pixel. Other small-scale patterns on the image are irrelevant to it. The LBGLCM approach extracts feature while taking into account every aspect of texture structure and spatial data. In this study, LBGLCM features from histopathological images are derived using the same formulas as the GLCM method.

When analyzing textures, scale is crucial information because the same texture can appear in many ways at various scales. The image pyramid, which is defined by sampling the image both in space and scale, is applied for expanding the LBGLCM to various scales in this study. It is often assumed that texture information is fixed at a particular image resolution in descriptors. If differing scales are taken into account when extracting texture descriptors from the photos, the texture descriptors’ ability to discriminate between objects can be considerably increased. The method for expanding LBGLCM to make it more resistant to scale change is presented in this paper.

The pyramid decomposition is used in this study to provide a variety of picture representations of the original image and suggests an extension of the LBGLCM to different scales. At every resolution level, LBP is produced, leading to the creation of several scales. A single feature descriptor is created by extracting the GLCM features from this generated LBP image at each scale. To benefit from the information from various scales, the features must be combined after being taken from each scale. A combination method is used to integrate the features in order to accomplish that. Each feature descriptor that is retrieved from a particular scale is normalized, and all of the normalized features are then concatenated to form a single set.

#### Color moment features

**Fig. 4:**
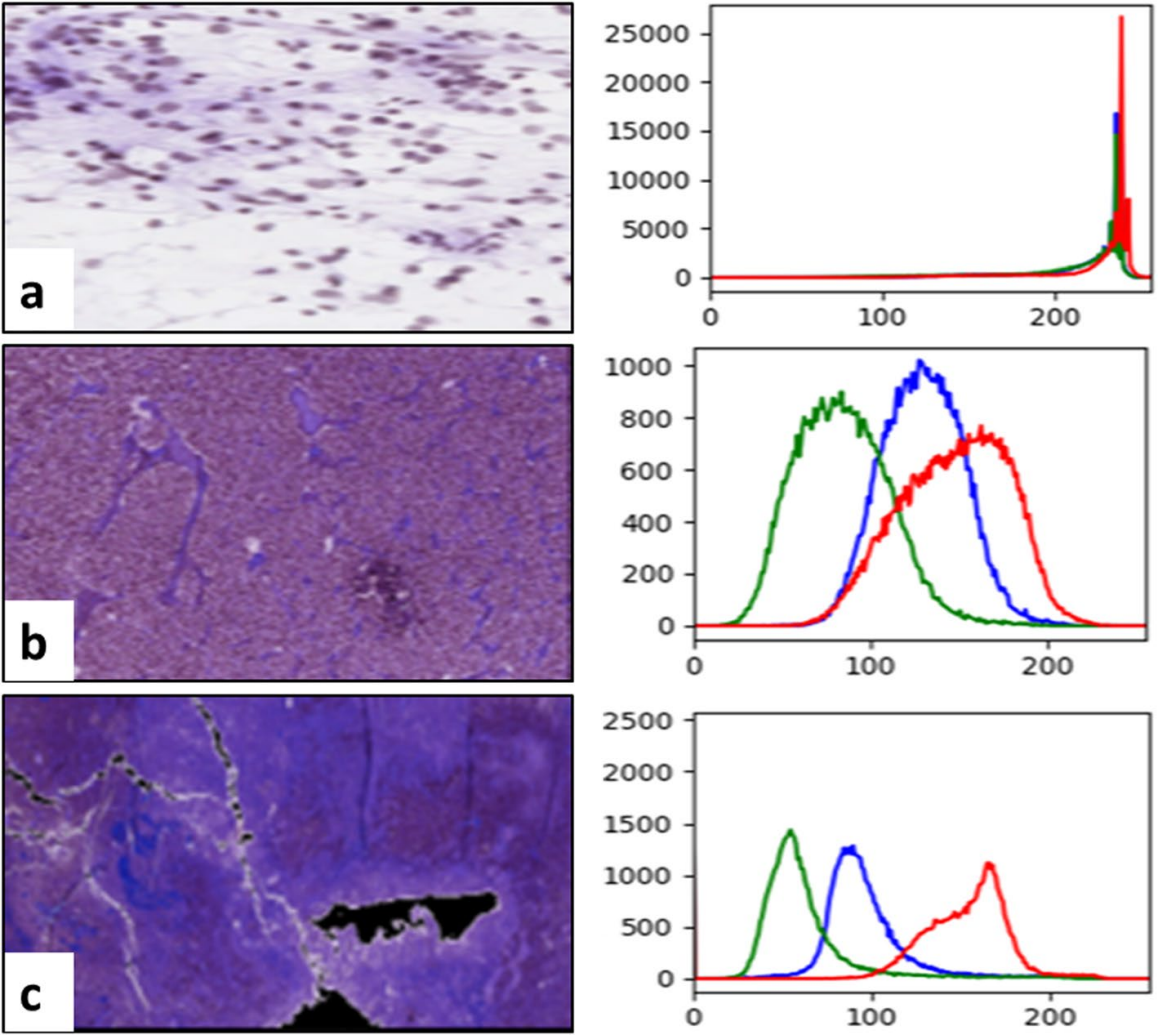
Brain biopsy images at a microscopic level: **a** Tissue image of grade II and color histogram, **b** Image of grade III and color histogram, and **c** Brain HI of grade IV and color histogram in RGB color space

The study of color moments was performed to obtain color-based characteristics from brain tissue pictures. A color histogram for the distribution of color in microscopic biopsy images is used for the color moment analysis. For the tissue image analysis, color information is highly significant, and each histogram peak represents another color, as illustrated by Fig. 4. The figure shows the number of color pixels present respectively in the image on the x-axis and y-axis of the color histogram.

**Fig. 5:**
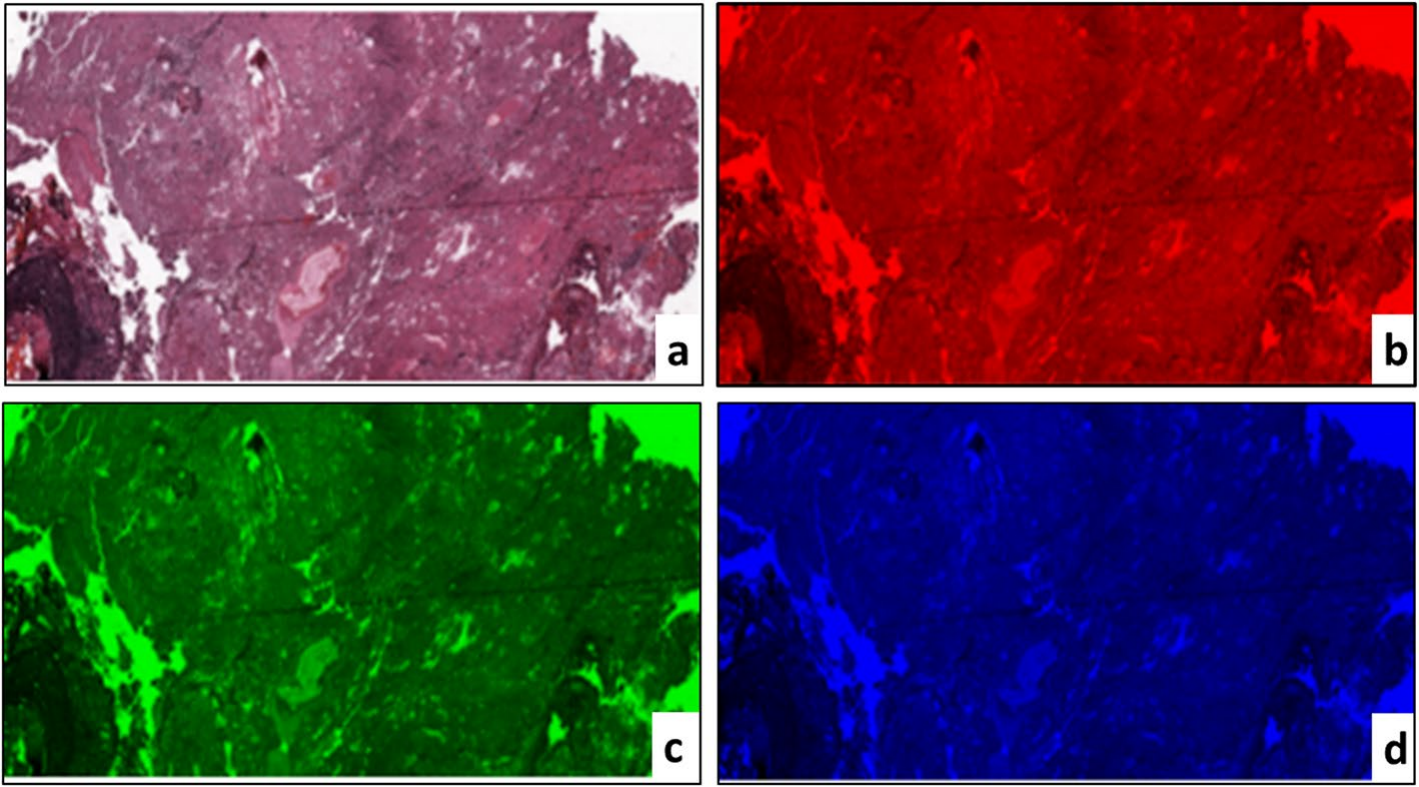
The HI color moment analyses by dividing RGB color channels: **a** Original RGB HI stained with H&E combination, **b** The Red component converted from an original, **c** The green component converted from an original, and **d** The blue component converted from an original

The PCMD technology was implemented to extract color-based information from images of the brain tissue. This method is helpful when the color distribution is analyzed between three RGB channels. The 3-color channels were split from RGB color images to obtain significative color moment information from tissue images, as shown in Fig. 5. Then, we independently calculated each channel’s mean, standard difference, skew, variance, and kurtosis. The following formulae were used to calculate these features.

18 $$\begin{aligned} Mean (\mu _{i}) = \sum \limits _{j=1}^{N} \frac{1}{N} \rho _{ij} \end{aligned}$$

19 $$\begin{aligned} Standard deviation(\sigma _{i}) = \sqrt{\frac{1}{N} \sum \limits _{1}^{N} (\rho _{ij} - \mu _{y})^{2} } \end{aligned}$$

20 $$\begin{aligned} Skewness (s_{i}) = \root 3 \of {\frac{1}{N} \sum \limits _{1}^{N} (\rho _{ij} - \mu _{y})^{3} } \end{aligned}$$

21 $$\begin{aligned} Kurtosis (k_{i}) = \root 4 \of {\frac{1}{N} \sum \limits _{1}^{N} (\rho _{ij} - \mu _{y})^{4} } \end{aligned}$$

where $p_{ij}$ is the $i^{th}$ pixel value of the color channel $i^{th}$ pixel picture. *N* is the pixel count in the picture. $\mu _{i}$ is the average, $\sigma _{i}$ is the default variation and is generated by the square root of the color distribution distinction, $s_{i}$ is the skewing value, and $k_{i}$ is the kurtosis value.

The HSV color space is utilized to reduce the influence of light variations. The component hue (*H*) has the color picture intense value, which is not altered by changes in lighting. The saturation (*S*) component also contrasts well with the processed picture [58].

**Rotation and scale invariant hybrid image descriptor (RSHD):** The RSHD method builds the descriptor over the entire image, which is viewed as a single region [59]. The RSHD is an effective merging of the color and texture cues that are already present in the image. The RGB color is quantized into a single channel with a smaller number of shades in order to encode the color information. The binary structuring pattern is used to compute the textural characteristic. By taking into account nearby local structure elements, structuring patterns are formed. The structural pattern is independently extracted for each quantized shade in order to combine the color and texture. This allows to simultaneously encode information about both color and texture. The local adjacent structure components make it easier for the suggested descriptor to gain rotation and scale-invariant properties. The RSHD divides the RGB color space into 64 hues. Utilizing 5 rotation invariant structure elements, the texture is also retrieved together with the color. In order to create an RSHD feature description, color and texture data are combined.

### Non-negative Matrix Factorization (NMF)-based feature reduction

After each pixel has generated the feature vector, the NMF approach is provided to minimize the dimensionality of the vector feature. NMF is a reduction approach on the basis of a low-rank feature space estimation as described by Eq. 22. It also ensures that the characteristics of the additives are non-negative [60].

22 $$\begin{aligned} x(i,j) \approx w(i,k)h(k,j) \end{aligned}$$

where *x* is a non-negative matrix representing the feature retrieved for every pixel in the processed image. *k* is a positive integer in which $k < (i,j)$. Two non-negative matrices, *w*(*i*, *k*) and *h*(*k*, *i*) are calculated. It reduces the standard of the x-wh differences. *w* can be seen as the reduced characteristics and *F* as the relevance of these characteristics. The product of *w* and *h* provides a reduced estimate of the data held within the *x* matrix. We have evaluated numerous *k* values to optimize the efficiency of the suggested system (from 2 to 100). We discovered that $k= 32$ performed best among other values.

### Classification

At this stage, a classifier is fed the reduced feature matrix in order to apply a mark matching a grade type for each pixel of the processed brain image. We would like to detect, as previously noted, there are four different levels of brain tumor, including oligodendroglioma, astrocytoma, oligoastrocytoma, and GBM.

The highly regarded random DT and SVM algorithms are the foundation of our ensemble classification approach. This choice results from a two-pronged strategy that builds diversity within the ensemble while leveraging each member’s unique strengths. Both random DT and SVM have gained recognition for their outstanding capabilities and resilience when dealing with complex classification tasks, especially those that involve high-dimensional feature spaces such as those found in histopathological image analysis. By using both random DT and SVM, we can take advantage of their complementary advantages and build a diverse and powerful ensemble that may result in higher classification accuracy for brain tumors.

#### Support vector machine

SVM is a powerful classification algorithm. SVM utilizes the kernel function to convert the original data space to a higher space. Eq. 23 defines the data separation hyper-plane function.

23 $$\begin{aligned} f(x_{i}) = \sum \limits _{n=1}^{N} \alpha _{n}y_{n}k(x_{n},x_{i}) + b \end{aligned}$$

where $x_{n}$ is supportive vector data (features from brain HI), $\alpha _{n}$ is a Lagrange, and $y_{n}$ is a goal class with $n=1, 2, 3, \ldots , N$. RBF kernel function is defined by Eq. 24.

24 $$\begin{aligned} k(x_{n},x_{i}) = \exp {(-\gamma \Vert {x_{n}-x_{i}}\Vert ^2 + C )} \end{aligned}$$

SVM contains *C* and $\gamma$, two main hyper-parameters. *C* is a hyper-parameter adjusting each support vector’s influence for the soft margin cost function. $\gamma$ is a hyper-parameter that determines the extent to which we desire the curvature at the decision limit. The values of $\gamma$ and *C* were set at [0.00001, 0.0001, 0.001] and [0.1, 1, 10, 100, 1000, 10000], respectively, and $\gamma$ and *C* were selected with maximum precision.

#### Fast large margin classifier

Soft edge SVMs can find hyper-planes with positive slack variables to adjust the constraints of Eq. 23. The slack variable adjustment allows SVM to lessen the influence of optimization by enabling certain situations to be within the margins or within another class. The concept of a large margin has been determined as a method for categorizing data based on the classification margin rather than as a raw training error. The categorization margin is primarily influenced by putting the decision-making function far away from any data points. Methods of fast marginal aim to reach wide marginal decision-making solutions through resolving a restricted quadratic issue of optimization and by promoting early stop approaches. The fast large classifier considers the above-mentioned optimization and convergence approaches to minimize errors and increase the hyper-planes’ separation margin. Such algorithms can help to optimize training procedures by saving time and resources. Let $\rho$ indicates the margin, how far two classes can be separated from each other, and therefore how quickly the learning algorithm has reached a point of convergence. The true mapping $f :x \rightarrow R$ can be used to classify pattern *x*, the margin may be determined using Eq. 25 [61]. It determines the function of margin costs $\vartheta ::R \rightarrow R^{+} and \vartheta$ risk of *f* given by Eq. 26.

25 $$\begin{aligned} \rho _{(f(x,y))} := yf(x) \end{aligned}$$

26 $$\begin{aligned} R_{\vartheta } (f)=E_{\vartheta \rho _{(f(x,y))}} \end{aligned}$$

The margin cost function of the AdaBoost algorithm $\vartheta (\alpha )= \exp (-\alpha )$. Classifiers must attain a broad margin $\rho _(f )$ for trustworthy training and should also be successful in unseen cases. The greatest margin *f* for the ideal hyper-plane can be provided to achieve the Eq. 27 between the weight vector and threshold.

27 $$\begin{aligned} w^{*} b^{*}= max_{i=1}^{m}min \frac{(w.x_i )+b}{\Vert w\Vert } \end{aligned}$$

By regulating the magnitude of *w* and the number of errors in training, we can minimize the following objective feature, in which the constant above 0, a properly generalized classifier can be obtained by Eq. 28.

28 $$\begin{aligned} \tau (w,\delta ) = \frac{1}{2}\Vert w\Vert ^2 + C\sum \limits _{i=1}^{m}\delta i \end{aligned}$$

#### Random decision tree

Random DT is one of the most widely used supervised ML techniques. The decisions are influenced by specific circumstances and can be interpreted easily. The key characteristics that are useful in classification are identified and chosen. It only selects attributes that return the biggest information gain (IG).

29 $$\begin{aligned} IG= E (Parent Node) - Average\; E(Child Nodes) \end{aligned}$$

where Entropy (*E*) is defined as: $E = \sum \nolimits _{i} - \;prob_{i}(log_2\; prob_i)$ and $prob_i$ is the probability of class *i*.

#### Majority-based voting mechanism

The majority vote is commonly applied in the ensemble classification. It is also referred to as voting for plurality. The proposed method uses a majority-based voting mechanism to enhance the classification results after implementing the three classification algorithms discussed above [62]. Each of these model results is calculated for each test case, and the final output is anticipated based on the majority of results. The *y* class mark is anticipated by majority votes for each classifier *C*, as presented by Eq. 30.

30 $$\begin{aligned} y \; = mod \{ C_{1}(x), C_{2}(x),....,C_{n}(x) \} \end{aligned}$$

## Experimental results

This section includes a description of the results, and discussion. The datasets are divided into training and testing sets in the Results section. The training set is then used to display the results of various feature extraction approaches using the NMF feature selection strategy. Then, using five performance measurements, we represent some tables and figures that support the desired idea. Finally, we objectively compare the proposed system with some literature research in the Discussion section.

### Results

Image augmentation expands the quantity of available data by applying domain-specific approaches to create altered versions of images. These transformations can include things like flips, zooms, and shifts, among other things. Image augmentation is distinguished from data preparation operations, such as image resizing and pixel scaling. Image augmentation is only used on the training dataset and not on the validation or test datasets. On the other hand, data preparation must be done consistently across all model datasets [63]. The data was then synthesized using a combination of affine image transformations, including rotation, shifting, scaling (zoom in/out), and flipping. We have evaluated a wide range of values for *k* for NMF feature reduction ($k = 2, 4, ...., 100$). In this case, *k* refers to the number of feature vector elements. We noticed that $k =32$ provides the best results for any glioma grades, reducing vector dimensions between 182 and 28 elements.

After preprocessing, 8,061 ROI from 821 different patients were used in our study for training and testing. To analyze the performance of our model, we specifically partitioned the dataset into 10 equal-sized folds and used 10-fold cross-validation. Approximately 7,255 ROIs from 739 patients made up 9/10 of the data in each fold, which was utilized for training, and approximately 806 ROIs from 82 patients made up the remaining 10% of the data, which was used for testing. Each fold was tested exactly once throughout each of the ten iterations of this procedure. This method allowed us to test our model’s performance and generalizability in a robust manner. With a 40% split between training and testing data, we also performed hold-out validation. Specifically, we used 40% of the data (about 3,224 ROIs from 328 patients) that were randomly chosen for testing, and the remaining 60% (about 4,837 ROIs from 493 patients) that were used for training.

**Fig. 6:**
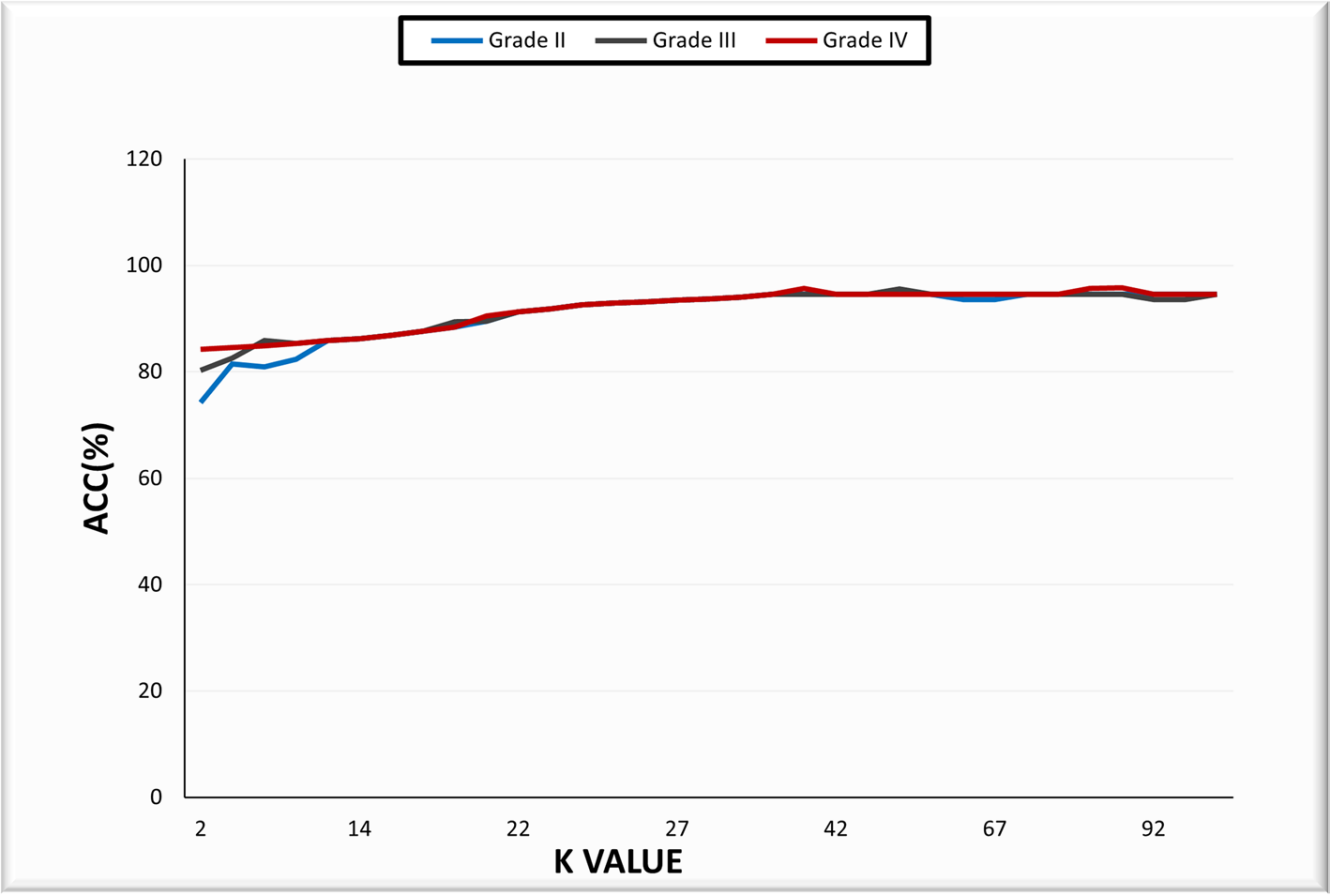
The accuracy obtained by experimenting with various k parameter values in the NMF technique

Figure 6 illustrates the ACC results in relation to the selection of various parameter *k* values for different brain glioma grades of the NMF approach. In order to evaluate the proposed method’s performance, we calculated the general ACC for the brain tumor classification. We compared the results with eight different state-of-the-art classifiers, namely K-nearest neighbor (KNN), generalized linear models (GLM), naive Bayesian ,RF, DT, SVM, gradient boosting trees (GBT), extreme gradient boosting (XGBoost) and DL classifier. In order to prevent overfitting, all results are collected using the 10-fold cross-validation technique. The results are shown in Table 4 for various models and different methods of features. The results of the accuracy of each brain glioma grade for 10-fold cross-validation are summarized in Table 5. The proposed system achieved an average overall accuracy of 95.8% for all tested images. Moreover, GLM, KNN, naive Bayesian, DT, RF, SVM,GBT,XGboost and DL classifier attained 92.1, 91%, 92.6%, 92.5%, 93.5%, 92.7, 93.7, 94.3, and 94.7%, respectively. The average accuracy of each glioma grade during holdout validation (40 percent for testing) is shown in Table 6. For all examined types of brain tumor images, the suggested approach had an overall average accuracy of 96%. While GLM, KNN, naive Bayesian, DT, RF, SVM,GBT,XGboost, and DL classifiers achieved 90.8%, 92%, 92.4%, 92.4%, 93.6%, 93.8%, 93.6%, 94.5%, and 94.8%, respectively. The results show that the proposed system, which is based on the hybrid ensemble technique, performs better than other state-of-the-art approaches.

**Table 4:** The comparison of the accuracy results for the different models and multiple methods of features

	KNN	GLM	Naïve Bayesian	RF	DT	GBT	XGboost	SVM	The Proposed classifier
GLCM	84.2	85.6	88.1	88.9	89.2	90	90.2	89.7	90.8
GLRLM	80.3	84.6	86.5	87.4	87.8	88.2	89	88.4	90.2
LBP	86.4	87.5	89	90.3	89.2	90.7	90.5	90.6	91.7
Multiple-LBGLCM	88	89.3	90.2	91.6	90.4	92	92.6	91.9	93.2
Color Moment Features	71.6	73.9	80.7	81.2	80.5	82.4	83.7	82.3	85.1
RSHD	88.4	88.8	90.4	91.2	89.7	92.3	92.8	90.6	92.7
Statistical methods (GLCM+ GLRLM)	85.5	89.4	88.6	90.5	90.6	90.9	91.3	90.2	91.8
Statistical methods + LBP	89.4	90.7	91.3	92.6	92.1	92.7	93	92.4	93.8
Statistical methods + Color Moment Features	85.7	87.2	88.3	89.4	90.4	90.1	90.7	90.8	91.8
LBP + Color Moment Features	87.2	89.1	89.8	91.3	90.8	91.5	91.8	91.2	92.4
Multiple-LBGLCM+ Color Moment Features	88	91.2	92.1	92.9	92.1	92.8	93.4	91.8	94.1
Statistical methods + LBP + Color Moment Features	90.6	91.4	91.3	91.1	92.8	93	93.2	93.3	94.6
The Proposed method	91	92.1	92.6	93.5	92.5	93.7	94.3	94	95.8

**Table 5:** The ACC (%) of different grades of tested brain tumor images by using NMF (k = 32) and 10-fold cross-validation

Classifiers	KNN	GLM	RF	DT	Naïve Bayesian	GBT	XGboost	SVM	DL classifier	The Proposed classifier
Grade II	91.4	92	93.7	92.8	93	93.8	94	94.2	94.8	96.2
Grade III	90.7	92.1	93.5	92.5	92.3	93.6	94.4	94	94.7	95.6
Grade IV	90.8	92.3	93.3	92.7	92.5	93.6	94.5	93.8	94.7	95.7
Average	91	92.1	93.5	92.6	92.6	93.7	94.3	92.7	94.7	95.8

**Table 6:** The ACC (%) of different grades of tested brain tumor images by using NMF (k = 32) and holdout validation technique (40% for testing)

Classifiers	KNN	GLM	RF	DT	Naïve Bayesian	GBT	XGboost	SVM	DL classifier	The Proposed
Grade II	91.2	91.8	93.8	92.6	92.8	93.6	94.4	94	94.9	96.4
Grade III	90.6	92.1	93.5	92.3	92.2	93.6	94.6	93.7	94.8	95.8
Grade IV	90.7	92.2	93.5	92.4	92.3	93.6	94.5	93.6	94.7	95.8
Average	90.8	92	93.6	92.4	92.4	93.6	94.5	93.8	94.8	96

**Fig. 7:**
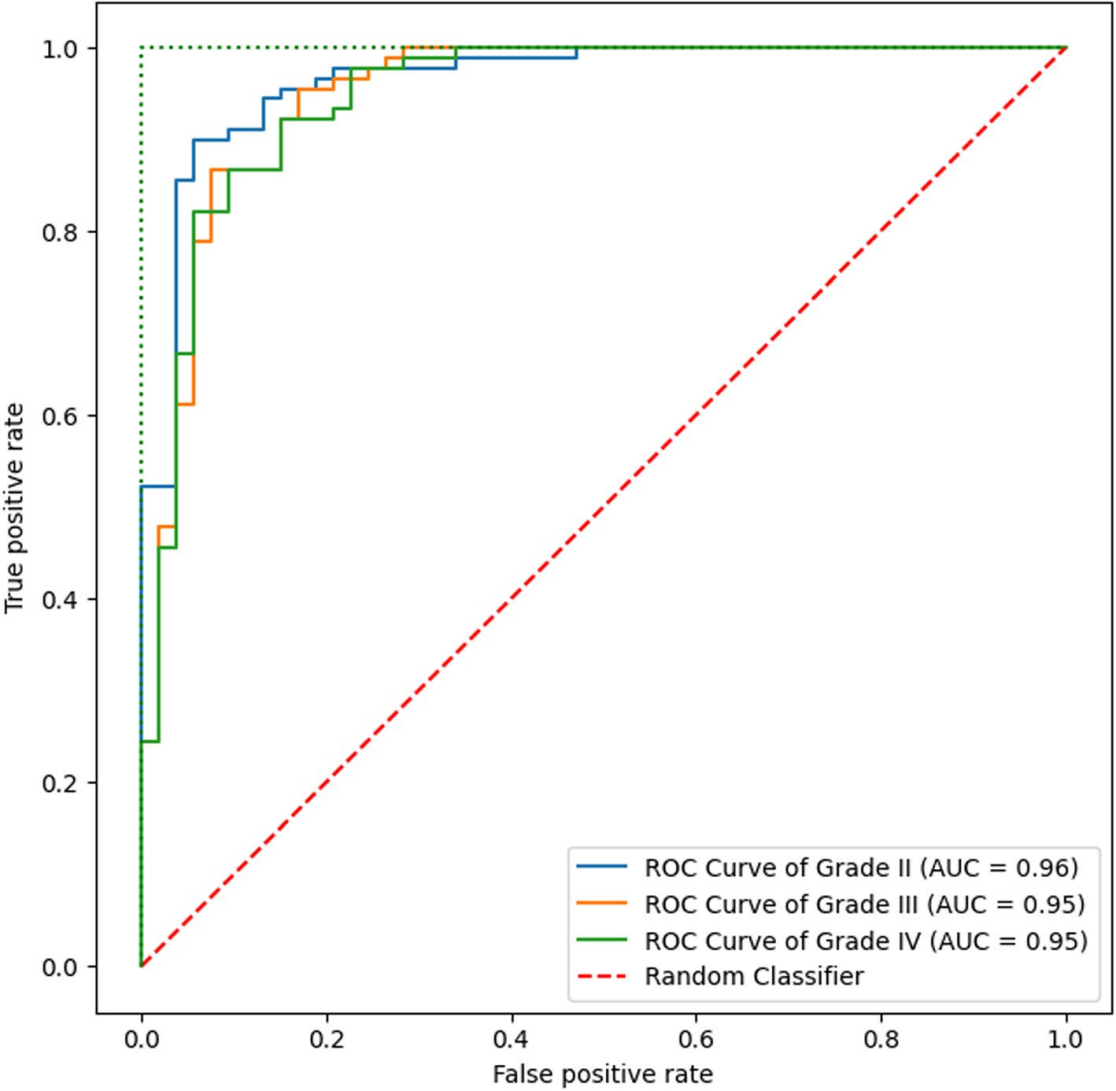
The area under the ROC curve for a multiclass brain tumor

By plotting the TPR against the false positive rate (FPR), the receiver operating characteristic (ROC) curve for the three respective classes is created. Figure 7 depicts the multiclass model’s relationship between sensitivity and specificity. For brain tumor grade, we determined the TPR and PPV. Table 4 presents the findings of PPV and TPR by the same value $k=32$ for NMF and with the 10-fold cross-validation technique. The table displays the system’s capacity to identify gliomas of various forms. We evaluated the performance of the proposed classification by comparing its results with three different state-of-the-art grade classification methods. The comparison with the state-of-the-art techniques is shown in Table 7.

**Table 7:** The comparison between the proposed system and state-of-the-art techniques

	Method	ACC(%)
Mousavi et al. [50]	Pseudopalisading Necrosis and MVP is identified using a combination of specific spatial and morphological filters with DT classifier.	84.7
Bhattacharjee et al.[28]	texture analysis was performed using Haar wavelet transformed GLCM, Color features was obtained using PCMD	91.7
Rathore et al. [31]	Utilizing a predictive classifier to evaluate the combined impact of clinical, textural, and traditional imaging aspects	90.4
Wang et al. [33]	Extract first-order and second-order features along with visual factors such as morphological parameters.	90
Durgamahanthi et al. [34]	Haralick features based on GLCM and GLRLM have been combined, and SVM has been utilized for classification.	93
The Proposed Method	A hybrid ensemble classification model was trained using a combination of color intensity and texturing features (GLCM, GLRLM, and LBP).	95.8

### Discussion

Recent advances and improvements in medical image tools have provided medical practitioners with ease and innovation. These developments help improve several fields of medicine, including disease detection, treatments, and prompt clinical application decision-making. Substantial medical information is generated daily in hospitals. Expert systems of clinical support are essential for the decision-making of health professionals. Medical computer research aids physicians and other professionals in their search for the finest possible solutions for taking advantage of these booming quantities of information.

Early identification and effective treatment options are needed to deal effectively with brain tumor illnesses. The treatment options depend on the tumor stage and the tumor grade when diagnosed. In the early phase of relevant feature extraction, conventional identification systems use some rudimentary machine-learning algorithms which extract only high-level and low-level capabilities. Here we provided a novel combination of profound and machine-learning methods for extracting and classifying functions. In the classification of brain tumors, the whole procedure was superior.

Our study aimed to distinguish between Grade II, III, and IV brain tumors, excluding Grade I tumors. These grades have complicated treatment requirements and fast growth rates, which present a serious clinical challenge. We could focus on features in the histopathological images that specifically distinguish these aggressive malignancies by eliminating Grade I tumors, which have a different biological behavior. By using this method, we were able to investigate further how well texture and color features distinguish the key characteristics that set these high-grade tumors apart, which may result in the development of more reliable classification models for better clinical decision-making.

The image can be viewed as a layout of various sections with varying color, texture, and shape. The neighborhood plays a significant impact in how the human visual system perceives the nearby structures since it provides a rich of texture and shape information. Structure elements have a significant impact on texture representation. However, these structural components are only somewhat rotationally invariant. The local adjacent structures of images belonging to the same class are quite similar, and if this information can be recorded in a rotation-invariant way, it can be useful for image classification. The current approach combines color and texture information to provide an image descriptor that is naturally rotation- and scale-invariant. To achieve this union, separate textural clues from each color are extracted.

The combination of structural and statistical methods used in LBP approaches increases the performance of texture analysis, and this is their most significant characteristic. The approach also has a simple implementation and a minimal computing cost. Additionally, it is unaffected by monotonic variations in illumination. The suggested method includes all structural information that was obtained using local binary patterns, and it also extracts additional information utilizing information of magnitude to increase discriminative power. The GLCM idea has been applied to extract the statistical characteristics for the categorization of texture images. By calculating the frequency of occurrence of identical patterns in various directions, GLCM directly interacts with the intensity of the images and offers the spatial relationship of the pixels in the image, making it beneficial for the extraction of texture characteristics.

Using the LBGLCM approach, features are extracted by taking into account every aspect of texture structure and spatial data. These benefits allow the LBGLCM method to outperform the other state of the arts algorithms in image analysis. Scale is crucial information for texture analysis because the same texture might seem differently at various scales. Multiple scales of the GLCM and LBP combination have been shown to be a very effective texture descriptor for use in feature extraction. Features that are based on color are regarded as being reliable and stable. They are not affected by changes in scale, rotation, or direction. In addition, the calculation of the color feature is a rather easy procedure. It is thought that HSV color space is appropriate for color description because of its properties. This is due to the fact that, for photographs acquired in diverse lighting situations, the image luminance is separated from the color information in the HSV color space, which might positively affect the further processing of these images.

**Fig. 8:**
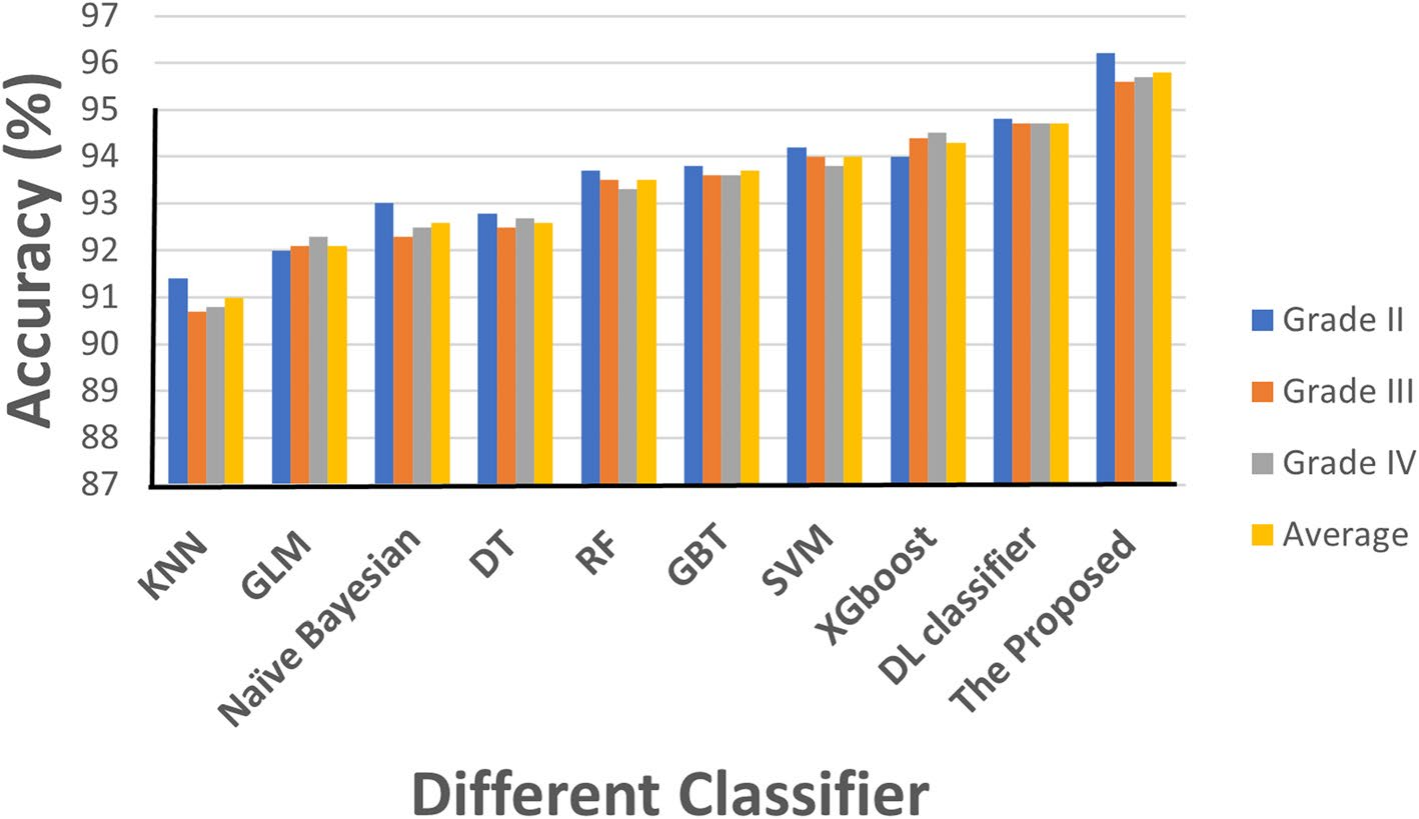
Comparison of the classifiers accuracy on the TCGA dataset

The proposed ensemble classifier performs best compared to many classifiers, such as SVM, KNN, naive baysean,DT, RF,GBT,XGboost,and DL classifier which are applied in this paper. In general, the goal was to improve performance using conventional classification systems. From Table 5, we can conclude that the proposed approach exceeds the comparative classifiers with ACC equals 95.8%. Figure 8 visulaize the giloma classification accuracy using different classifiers which indicates the proposed classifier with the highest performance. In the hybrid ensemble classification system, we utilized the RBF-SVM, fast large margin, and DT since they deliver the greatest performance in many evaluation criteria compared to others. Sensitivity results indicate the good determinative tumors that are benign since they are only a positive total division between the genuine total benign tumor. Specific results are good determinatives for malignant tumors since it is just a total negative division between total actual malignant tumors. DSC describes the equilibrium between precision and sensitivity, which is essential for unequal class distribution. Precision describes the actual TP percentage, which is rather high for the suggested approach, of the full projected positive TP+FP. Overall, Table 7 compare the performance of the proposed classification methodology with the recently state of the arts.

## Conclusion and future work

Histopathology images are commonly utilized for diagnosing the brain and determining cancer malignancy. Biopsied tissue is stained with H&E and inspected under the microscope by pathologists to identify the histological grades of the brain. This work aims to develop an automated glioma grading platform based on ML models. This study’s main objective is to assess the complicated random field of cancer images with color intensity (color moment features) and texture features (GLCM, LBP, multi-LBGLCM, GLRLM, and RSHD) and the fusion of various features. Using different methods for texture features, we could get helpful information from histology pictures to predict cancer grades. In the proposed method, a hybrid ensemble classification was used. All the procedures required for analyzing, extracting, and classifying photos of biopsies were implemented, which performed well in brain tissue images. The proposed method shows a high accuracy when compared favorably to several state-of-the-art techniques. The results show that the suggested approach can be useful to a pathologist in efficiently classifying histopathological slides into LGG and HGG categories, especially when examining a large number of slides is required. Although this study concentrated on using color and texture features for analysis, In future work, we will try to include other morphological features as well, which could provide a deeper comprehension of the image data. In the process, we hope to advance deep learning for brain tumor classification and develop more reliable and accurate diagnostic tools. Also, Future studies aim to expand the scope of this work beyond glioma classification by including diverse medical image datasets (skin, breast, and lung).

## Data Availability

The datasets used during the current study available in the Cancer Genome Atlas repository (https://portal.gdc.cancer.gov/) at the TCGA-LGG and TCGA-GBM projects.

## Declarations

This research study was conducted retrospectively using human subject data made available in open access by: https://portal.gdc.cancer.gov/projects/TCGA-GBM and https://portal.gdc.cancer.gov/projects/TCGA-LGG.

## References

[1] Tumour B. Brain Tumor: Diagnosis. https://www.cancer.net/cancer-types/brain-tumor/diagnosis. Accessed 18 May 2024.

[2] Brain. Cancer. https://www.who.int/health-topics/cancer. Accessed 18 May 2024.

[3] Charity BT. The Brain Tumour Charity. https://www.thebraintumourcharity.org. Accessed 18 May 2024.

[4] Shah V, Kochar P. Brain Cancer: Implication to Disease, Therapeutic Strategies and Tumor Targeted Drug Delivery Approaches. Recent Patents Anti Cancer Drug Discov. 2018;13(1):70–85. 10.2174/1574892812666171129142023 29189177

[5] Ayyad SM, Shehata M, Shalaby A, El-Ghar M, Ghazal M, El-Melegy M, et al. Role of AI and Histopathological Images in Detecting Prostate Cancer: A Survey. Sensors (Basel, Switzerland). 2021;21(8):2586. 10.3390/s21082586 PMC8067693 33917035

[6] Hsu W, Guo JM, Pei L, Chiang LA, Li YF, Hsiao JC, et al. A weakly supervised deep learning-based method for glioma subtype classification using WSI and mpMRIs. Sci Rep. 2022;12. 10.1038/s41598-022-09985-1 PMC9005548 35414643

[7] Shajahan S, Pathmanaban S, Tiruvenkadam K. RIBM3DU-Net: Glioma tumour substructures segmentation in magnetic resonance images using residual-inception block with modified 3D U-Net architecture. Int J Imaging Syst Technol. 2024;34(2):e23056. 10.1002/ima.23056

[8] Shirazi AZ, Fornaciari E, Bagherian NS, Ebert L, Koszyca B, Gomez GA. DeepSurvNet: deep survival convolutional network for brain cancer survival rate classification based on histopathological images. Med Biol Eng Comput. 2020;58:1031–45. 32124225 10.1007/s11517-020-02147-3 PMC7188709

[9] Perrin SL, Samuel M, Koszyca B, Brown M, Ebert L, Oksdath M, et al. Glioblastoma heterogeneity and the tumour microenvironment: implications for preclinical research and development of new treatments. Biochem Soc Trans. 2019;47(2):625–38. 30902924 10.1042/BST20180444

[10] Griffin J, Treanor D. Digital pathology in clinical use: where are we now and what is holding us back? Histopathology. 2017;70(1):134–45. 27960232 10.1111/his.12993

[11] Yonekura A, Kawanaka H, Prasath VBS, Aronow B, Tsuruoka S. Glioma Subtypes Clustering Method using Histopathological Image Analysis. In: 2018 Joint 7th International Conference on Informatics, Electronics & Vision (ICIEV) and 2018 2nd International Conference on Imaging, Vision & Pattern Recognition (icIVPR). Kitakyushu: IEEE; 2018. pp. 442–6.

[12] Zhou L, Zhang Z, Chen YC, Zhao Z, Yin X, Jiang H. A Deep Learning-Based Radiomics Model for Differentiating Benign and Malignant Renal Tumors1. Transl Oncol. 2019;12:292–300. 30448734 10.1016/j.tranon.2018.10.012 PMC6299150

[13] TCGA. The Cancer Genome Atlas, TCGA-GBM, TCGA-LGG. https://portal.gdc.cancer.gov/repository. Accessed 18 May 2024.

[14] de Oliveira CI, do Nascimento MZ, Roberto GF, Tosta TA, Martins AS, Neves LA. Hybrid models for classifying histological images: An association of deep features by transfer learning with ensemble classifier. Multimed Tools Appl. 2024;83(8):21929–52. 10.1007/s11042-023-16351-4

[15] Siegel RL, Miller KD, Goding Sauer A, Fedewa SA, Butterly LF, Anderson JC, et al. Colorectal cancer statistics, 2020. CA Cancer J Clin. 2020;70(3):145–64. 32133645 10.3322/caac.21601

[16] Xu H, Park S, Hwang TH. Computerized classification of prostate cancer gleason scores from whole slide images. IEEE/ACM Trans Comput Biol Bioinforma. 2019;17(6):1871–82. 10.1109/TCBB.2019.2941195 31536012

[17] Liu XP, Jin X, Seyed Ahmadian S, Yang X, Tian SF, Cai YX, et al. Clinical significance and molecular annotation of cellular morphometric subtypes in lower-grade gliomas discovered by machine learning. Neuro Oncol. 2023;25(1):68–81. 35716369 10.1093/neuonc/noac154 PMC9825346

[18] Li X, Li C, Rahaman MM, Sun H, Li X, Wu J, et al. A comprehensive review of computer-aided whole-slide image analysis: from datasets to feature extraction, segmentation, classification and detection approaches. Artif Intell Rev. 2022;55(6):4809–78. 10.1007/s10462-021-10121-0

[19] Krithiga R, Geetha P. Breast cancer detection, segmentation and classification on histopathology images analysis: a systematic review. Arch Comput Methods Eng. 2021;28:2607–19. 10.1007/s11831-020-09470-w

[20] Chang H, Nayak NM, Spellman P, Parvin B. Characterization of Tissue Histopathology via Predictive Sparse Decomposition and Spatial Pyramid Matching. Med Image Comput Comput Assist Interv MICCAI Int Conf Med Image Comput Comput Assist Interv. 2013;16(Pt 2):91–8. 10.1007/978-3-642-40763-5_12 PMC3998828 24579128

[21] TCGA. Glioblastoma Multiforme from TCGA. https://wiki.cancerimagingarchive.net/display/Public/TCGA-GBM. Accessed 18 May 2024.

[22] TCGA. Kidney Renal Clear Cell Carcinoma from TCGA. https://portal.gdc.cancer.gov/repository. Accessed 18 May 2024.

[23] Alberts E, Tetteh G, Trebeschi S, Bieth M, Valentinitsch A, Wiestler B, et al. Multi-modal Image Classification Using Low-Dimensional Texture Features for Genomic Brain Tumor Recognition. In: GRAIL/MFCA/MICGen@MICCAI. Québec City: Springer; 2017.

[24] Amin J, Sharif M, Raza M, Yasmin M. Detection of Brain Tumor based on Features Fusion and Machine Learning. J Ambient Intell Humanized Comput. 2018;15:1–17.

[25] Virupakshappa Amarapur B. Computer-aided diagnosis applied to MRI images of brain tumor using cognition based modified level set and optimized ANN classifier. Multimed Tools Appl. 2018;79:3571–99. 10.1007/s11042-018-6176-1

[26] Barker J, Hoogi A, Depeursinge A, Rubin D. Automated classification of brain tumor type in whole-slide digital pathology images using local representative tiles. Med Image Anal. 2016;30:60–71. 26854941 10.1016/j.media.2015.12.002

[27] Powell RT, Olar A, Narang S, Rao G, Sulman E, Fuller G, et al. Identification of Histological Correlates of Overall Survival in Lower Grade Gliomas Using a Bag-of-words Paradigm: A Preliminary Analysis Based on Hematoxylin & Eosin Stained Slides from the Lower Grade Glioma Cohort of The Cancer Genome Atlas. J Pathol Inform. 2017;8(1):9. 10.4103/jpi.jpi_43_16 PMC5364741 28382223

[28] Bhattacharjee S, Kim CH, Park HG, Prakash D, Madusanka N, Cho N, et al. Multi-Features Classification of Prostate Carcinoma Observed in Histological Sections: Analysis of Wavelet-Based Texture and Colour Features. Cancers. 2019;11(12):1937. 10.3390/cancers11121937 PMC6966617 31817111

[29] Rathore S, Iftikhar MA, Chaddad A, Niazi T, Karasic T, Bilello M. Segmentation and Grade Prediction of Colon Cancer Digital Pathology Images Across Multiple Institutions. Cancers. 2019;11(11):1700. 10.3390/cancers11111700 PMC6896042 31683818

[30] Chiesa-Estomba C, Echaniz O, Larruscain E, González-García J, Sistiaga-Suárez J, Graña M. Radiomics and Texture Analysis in Laryngeal Cancer. Looking for New Frontiers in Precision Medicine through Imaging Analysis. Cancers. 2019;11(10):1409. 10.3390/cancers11101409 PMC6826870 31547210

[31] Rathore S, Niazi T, Iftikhar MA, Chaddad A. Glioma grading via analysis of digital pathology images using machine learning. Cancers. 2020;12(3):578. 32131409 10.3390/cancers12030578 PMC7139732

[32] Hemanth G, Janardhan M, Sujihelen L. Design and Implementing Brain Tumor Detection Using Machine Learning Approach. In: 2019 3rd International Conference on Trends in Electronics and Informatics (ICOEI). 2019. pp. 1289–94.

[33] Wang X, Wang D, Yao Z, Xin B, jie Wang B, Lan C, et al. Machine Learning Models for Multiparametric Glioma Grading With Quantitative Result Interpretations. Front Neurosci. 2019;12. 10.3389/fnins.2018.01046 PMC6337068 30686996

[34] Durgamahanthi V, Anita Christaline J, Shirly Edward A. GLCM and GLRLM Based Texture Analysis: Application to Brain Cancer Diagnosis Using Histopathology Images. In: Intelligent Computing and Applications. Singapore: Springer Singapore; 2021. pp. 691–706.

[35] Sikder J, Das UK, Chakma RJ. Supervised learning-based cancer detection. Int J Adv Comput Sci Appl. 2021;12(5):863-9.

[36] Ahmad N, Asghar S, Gillani SA. Transfer learning-assisted multi-resolution breast cancer histopathological images classification. Vis Comput. 2022;38:2751–70. 10.1007/s00371-021-02153-y

[37] Xiao R, Debreuve E, Ambrosetti D, Descombes X. Renal Cell Carcinoma Classification from Vascular Morphology. In: MICCAI. Strasbourg: Springer; 2021.

[38] Dasanayaka S, Shantha V, Silva S, Meedeniya DA, Ambegoda TD. Interpretable machine learning for brain tumour analysis using MRI and whole slide images. Softw Impacts. 2022;13:100340. 10.1016/j.simpa.2022.100340

[39] Myronenko A. 3D MRI brain tumor segmentation using autoencoder regularization. In: BrainLes@MICCAI. Granada: Springer; 2018.

[40] Attallah O, Zaghlool SB. AI-Based Pipeline for Classifying Pediatric Medulloblastoma Using Histopathological and Textural Images. Life. 2022;12(2):232. 10.3390/life12020232 PMC8879027 35207519

[41] Ker J, Bai Y, Lee HY, Rao JP, Wang L. Automated brain histology classification using machine learning. J Clin Neurosci. 2019;66:239–45. 31155342 10.1016/j.jocn.2019.05.019

[42] Rinesh S, Maheswari KU, Arthi B, Sherubha P, Vijay A, Sridhar S, et al. Investigations on Brain Tumor Classification Using Hybrid Machine Learning Algorithms. J Healthc Eng. 2022;2022(1):2761847. 10.1155/2022/2761847 PMC8860516 35198132

[43] Zhou X, Tang C, Huang P, Tian S, Mercaldo F, Santone A. ASI-DBNet: An Adaptive Sparse Interactive ResNet-Vision Transformer Dual-Branch Network for the Grading of Brain Cancer Histopathological Images. Interdiscip Sci Comput Life Sci. 2022;15:15–31. 10.1007/s12539-022-00532-0 35810266

[44] Khan MA, Khan A, Alhaisoni MM, Alqahtani A, Alsubai S, Alharbi M, et al. Multimodal brain tumor detection and classification using deep saliency map and improved dragonfly optimization algorithm. Int J Imaging Syst Technol. 2022;33:572–87. 10.1002/ima.22831

[45] Syedsafi S, Sriramakrishnan P, Kalaiselvi T. An Automated Two-Stage Brain Tumour Diagnosis System Using SVM and Geodesic Distance-Based Colour Segmentation. In: International Conference on Power Engineering and Intelligent Systems (PEIS). Singapore: Springer; 2023. pp. 179–91.

[46] Khan F, Gulzar Y, Ayoub S, Majid M, Mir MS, Soomro AB. Least Square-Support Vector Machine Based Brain Tumor Classification System with Multi Model Texture Features. Front Appl Math Stat. 2023;9:1324054. 10.3389/fams.2023.1324054

[47] Gül M, Kaya Y. Comparing of brain tumor diagnosis with developed local binary patterns methods. Neural Computing and Applications. 2024;36:1–14.

[48] Nanda A, Barik RC, Bakshi S. SSO-RBNN driven brain tumor classification with Saliency-K-means segmentation technique. Biomed Signal Process Control. 2023;81:104356. 10.1016/j.bspc.2022.104356

[49] Saba T, Mohamed AS, El-Affendi M, Amin J, Sharif M. Brain tumor detection using fusion of hand crafted and deep learning features. Cogn Syst Res. 2020;59:221–30. 10.1016/j.cogsys.2019.09.007

[50] Mousavi H, Monga V, Rao G, Rao A. Automated discrimination of lower and higher grade gliomas based on histopathological image analysis. J Pathol Inform. 2015;6(1):15. 10.4103/2153-3539.153914 PMC4382761 25838967

[51] Tosta TAA, de Faria FR, Neves LA, do Nascimento MZ. Computational normalization of H &E-stained histological images: Progress, challenges and future potential. Artif Intell Med. 2019;95:118–32. 30420242 10.1016/j.artmed.2018.10.004

[52] Mobadersany P, Yousefi S, Amgad M, Gutman D, Barnholtz-Sloan J, Vega JEV, et al. Predicting cancer outcomes from histology and genomics using convolutional networks. Proc Natl Acad Sci USA. 2018;115:E2970–9. 29531073 10.1073/pnas.1717139115 PMC5879673

[53] Kumar N, Verma R, Sharma S, Bhargava S, Vahadane A, Sethi A. A Dataset and a Technique for Generalized Nuclear Segmentation for Computational Pathology. IEEE Trans Med Imaging. 2017;36:1550–60. 28287963 10.1109/TMI.2017.2677499

[54] Sriramakrishnan P, Kalaiselvi T, Rajeswaran R. Modified local ternary patterns technique for brain tumour segmentation and volume estimation from MRI multi-sequence scans with GPU CUDA machine. Biocybernetics Biomed Eng. 2019;39(2):470–87. 10.1016/j.bbe.2019.02.002

[55] Kaplan K, Kaya Y, Kuncan M, Ertunç HM. Brain tumor classification using modified local binary patterns (LBP) feature extraction methods. Med Hypotheses. 2020;139:109696. 32234609 10.1016/j.mehy.2020.109696

[56] Erfankhah H, Yazdi M, Babaie M, Tizhoosh H. Heterogeneity-Aware Local Binary Patterns for Retrieval of Histopathology Images. IEEE Access. 2019;7:18354–67. 10.1109/ACCESS.2019.2897281

[57] Jurio A, Bustince H, Pagola M, Couto P, Pedrycz W. New measures of homogeneity for image processing: an application to fingerprint segmentation. Soft Comput. 2014;18:1055–66. 10.1007/s00500-013-1126-3

[58] Zarella M, Breen D, Plagov A, Garcia FU. An optimized color transformation for the analysis of digital images of hematoxylin & eosin stained slides. J Pathol Inform. 2015;6(1):33. 10.4103/2153-3539.158910 PMC4485192 26167377

[59] Dubey SR, Singh SK, Singh RK. Rotation and scale invariant hybrid image descriptor and retrieval. Comput Electr Eng. 2015;46:288–302. 10.1016/j.compeleceng.2015.04.011

[60] Gillis N. The why and how of nonnegative matrix factorization. Regularization, Optimization, Kernels, and Support Vector Mach. 2014;12(257):257-91.

[61] Ibraheem MR, Adel J, Balbaa AEA, El-Sappagh S, Abuhmed T, Elmogy MM. Timing and Classification of Patellofemoral Osteoarthritis Patients Using Fast Large Margin Classifier. Cmc Comput Mater Continua. 2021;67:393–409.

[62] Kang X, Lin G, Jun Chen Y, Zhao F, Zhang E, Jing C. Robust and secure zero-watermarking algorithm for color images based on majority voting pattern and hyper-chaotic encryption. Multimed Tools Appl. 2019;79:1169–202. 10.1007/s11042-019-08191-y

[63] Zouggar ST, Adla A. Optimization techniques for machine learning. In: Optimization in Machine Learning and Applications. Singapore: Springer; 2020. pp. 31–50.

